# Evolution of a G protein-coupled receptor response by mutations in regulatory network interactions

**DOI:** 10.1038/ncomms12344

**Published:** 2016-08-04

**Authors:** Raphaël B. Di Roberto, Belinda Chang, Ala Trusina, Sergio G. Peisajovich

**Affiliations:** 1 Department of Cell and Systems Biology, University of Toronto, 25 Harbord Street, Toronto, Ontario, Canada M5S 3G5; 2 Niels Bohr Institute, University of Copenhagen, Blegdamsvej 17, Copenhagen Ø 2100, Denmark

## Abstract

All cellular functions depend on the concerted action of multiple proteins organized in complex networks. To understand how selection acts on protein networks, we used the yeast mating receptor Ste2, a pheromone-activated G protein-coupled receptor, as a model system. In *Saccharomyces cerevisiae*, Ste2 is a hub in a network of interactions controlling both signal transduction and signal suppression. Through laboratory evolution, we obtained 21 mutant receptors sensitive to the pheromone of a related yeast species and investigated the molecular mechanisms behind this newfound sensitivity. While some mutants show enhanced binding affinity to the foreign pheromone, others only display weakened interactions with the network's negative regulators. Importantly, the latter changes have a limited impact on overall pathway regulation, despite their considerable effect on sensitivity. Our results demonstrate that a new receptor–ligand pair can evolve through network-altering mutations independently of receptor–ligand binding, and suggest a potential role for such mutations in disease.

## Abstract (web summary)

Co-evolution of a new receptor-ligand pair will affect the downstream signal transduction network. Here, the authors use experimental evolution of yeast mating receptor Ste2 to show the effect of enhanced binding affinity and weakened interactions with the network's negative regulators on protein evolution.

## Graphical abstract

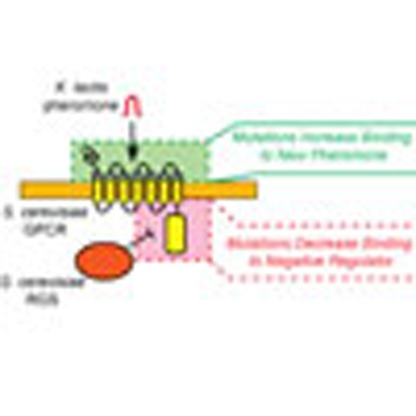

Important cellular processes result from the concerted action of multiple proteins organized in complex networks. Studies in evolution have revealed how individual proteins can acquire new functions due to changes in their binding specificity or catalytic potential^1^,^2^,^3^,^4^,^5^. However, these characteristics alone often cannot explain the evolution of complex cellular functions, because network output does not solely depend on the function of an individual protein, but rather on the integrated function of multiple components with intricate regulatory relationships^6^.

Past work has found evidence of network ‘re-wiring' in evolution from large-scale data^7^,^8^,^9^, whereby proteins are conserved across organisms but the interactions between them differ, although the molecular basis of such re-wiring is not always evident. Studies focusing on well-characterized signalling systems in bacteria^10^, yeast^11^ and mammals^12^,^13^ have shed light on how changes in protein interactions can alter regulatory networks. Similarly, recent work has demonstrated that domain shuffling can extensively re-wire a signalling network by exchanging interaction motifs between proteins^14^,^15^,^16^, a process that has been linked to the evolution of organism complexity^17^. Though much progress has been made, it remains unclear how selection acts on regulatory networks during the evolution of a new function.

To examine this question, we followed in real time the initial steps in the evolution of the response to a new ligand in the yeast pheromone receptor Ste2, a G protein-coupled receptor (GPCR). In *Saccharomyces cerevisiae* (hereafter abbreviated *Scer*) and in related ascomycetes, the mating process involves the fusion of two haploid cells to produce a diploid daughter^18^. In one mating type, this process is triggered when the pheromone α-factor binds to its cognate receptor Ste2. This GPCR acts as a network hub, mediating both signal transduction and signal suppression through interactions with multiple protein partners (Fig. 1a). These interactions have been extensively studied and are conserved from yeast to humans^19^,^20^,^21^. For signal transduction, pheromone-bound Ste2 mediates the exchange of GDP for GTP in a heterotrimeric G protein. This triggers a mitogen-activated protein kinase (MAPK) cascade leading to the expression of mating-related genes. In addition, Ste2 contributes to signal suppression through its cytoplasmic domain. First, the receptor brings Sst2, a regulator of G-protein signalling (RGS), in proximity to its target, Gpa1_α_, to promote GTP hydrolysis and shut down signalling^22^. Second, Ste2 is internalized as homo-oligomers and degraded both constitutively and on ligand binding, a process that involves sequential receptor phosphorylation, ubiquitination and the recruitment of the clathrin endocytic machinery^23^,^24^,^25^,^26^. Ste2's central role in both network activation and signal suppression, along with its well-characterized and highly conserved regulatory interactions, make it an ideal target to investigate the contribution of network-altering mutations to the evolution of new function.

We hypothesized that the evolution of a new function in the yeast mating pathway could occur through changes in the signalling hub Ste2. To test this, we mimicked an evolutionary scenario in which *Scer* cells were under selection pressure to respond to a weak agonist, the pheromone of the related species *Kluyveromyces lactis* (hereafter abbreviated *Klac*). To focus on the receptor and its interactions, we introduced random mutations only in Ste2. We then used high-throughput selection to isolate cells that activated the mating pathway in response to *Klac* α-factor. We investigated the contribution of network-altering mutations by performing a detailed phenotypic analysis on a subset of selected variants.

Our analysis revealed two distinct evolutionary paths: a ‘classical' path involving improvements in binding affinity for the foreign agonist; and a ‘network-altering' path, wherein the interaction between the receptor and the RGS is no longer conducive to signal suppression due to a partial loss of the receptor's cytoplasmic tail. Importantly, receptors truncations have only a limited effect on pathway regulation, suggesting that the partial loss of this interaction-rich region can be an acceptable evolutionary strategy, an observation supported by the large variability in cytoplasmic tail lengths found among Ste2 homologues. Altogether, these results point to a novel mechanism of network evolution, and suggest a possible link between RGS proteins and disease-causing GPCR mutations.

## Results

### Directed evolution of Ste2 yields diverse response profiles

**Figure 1:**
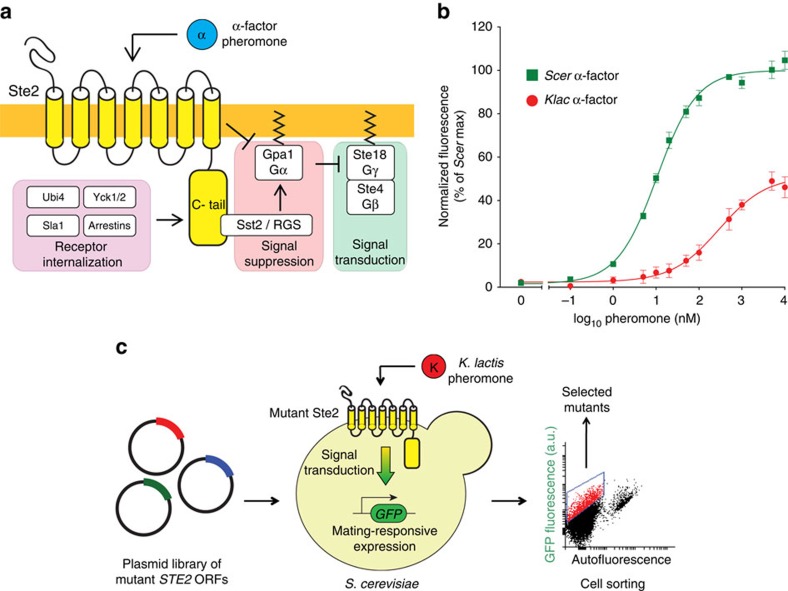
The G protein-coupled receptor Ste2, a network hub in the yeast mating pathway, is used to study the contribution of network interactions in the evolution of a new response. (**a**) Ste2 contributes to both signal transduction and signal suppression through a variety of physical interactions. (**b**) Dose-response relationship of cells expressing GFP in response to mating pathway activation. Cells were treated with various concentrations of pheromone and incubated for 3 h. GFP fluorescence was measured by flow cytometry. Wild-type Ste2 confers a weak, low-sensitivity response to *Klac* α-factor, a foreign pheromone. Error bars represent the s.e.m. (**c**) Schematic view of our directed evolution method. A plasmid library of *STE2* mutants generated by error-prone PCR was transformed in *ste2*Δ yeast. Cells were treated with 5 μM *Klac* α-factor and incubated for 3 h. Fluorescence-activated cell sorting was used to select for highly-activating variants which were then plated. Individual colonies were screened to confirm the desired phenotype and sequenced.

To characterize the mating response of *Scer* cells with different pheromones, we used a strain in which the promoter of the gene *FUS1* drives the expression of green fluorescent protein (GFP)^27^. We found that wild-type *Scer* cells respond weakly but consistently to *Klac* α-factor with a lower sensitivity (higher EC50) and a lower maximum than the response to *Scer* α-factor (Fig. 1b). We also tested the α-factor pheromone of two more related species: *Naumovozyma castellii* (formerly *Saccharomyces castellii*) and *Candida glabrata* (abbreviated *Cgla*). However, the former elicited a response comparable to that of the native *Scer* pheromone while the response to the latter was negligible (data not shown). As we sought a weak, but measurable response, we proceeded to use *Klac* α-factor for our directed evolution experiment.

We used directed evolution to obtain variants of the pheromone receptor Ste2 that conferred a strong response to *Klac* α-factor (Fig. 1c). First, we transformed a *ste2*Δ yeast strain with a plasmid-based library of *STE2* mutants generated by error-prone PCR. We then used fluorescence-activated cell sorting to select cells able to respond strongly to treatment with 5 μM *Klac* α-factor. After two iterative rounds of cell sorting followed by a screening step to isolate individual non-constitutive variants, we obtained 21 mutant receptors capable of responding strongly to *Klac* α-factor.

Sequencing of the selected Ste2 variants revealed a diversity of genotypes with one or more protein mutations (Supplementary Table 1), and mutated sites spread throughout the entire receptor (Supplementary Fig. 1A). The mutant receptors were labelled according to their most severe protein mutation (S: substitution, T: truncation, F: frameshift) and numbered. Many of the mutated sites were recurrent within our set of selected variants, or had been implicated in receptor function in past studies^24^,^28^,^29^,^30^,^31^,^32^,^33^,^34^,^35^,^36^,^37^,^38^,^39^.

We found that all selected receptors retained their ability to respond strongly to *Scer* pheromone, with most also displaying the ability to respond to *Cgla* α-factor (Supplementary Fig. 1B). These two features, a robust native response and the facile emergence of promiscuity in the function under selection, are thought to underlie the evolution of new protein functions in nature^40^,^41^,^42^,^43^.

**Figure 2:**
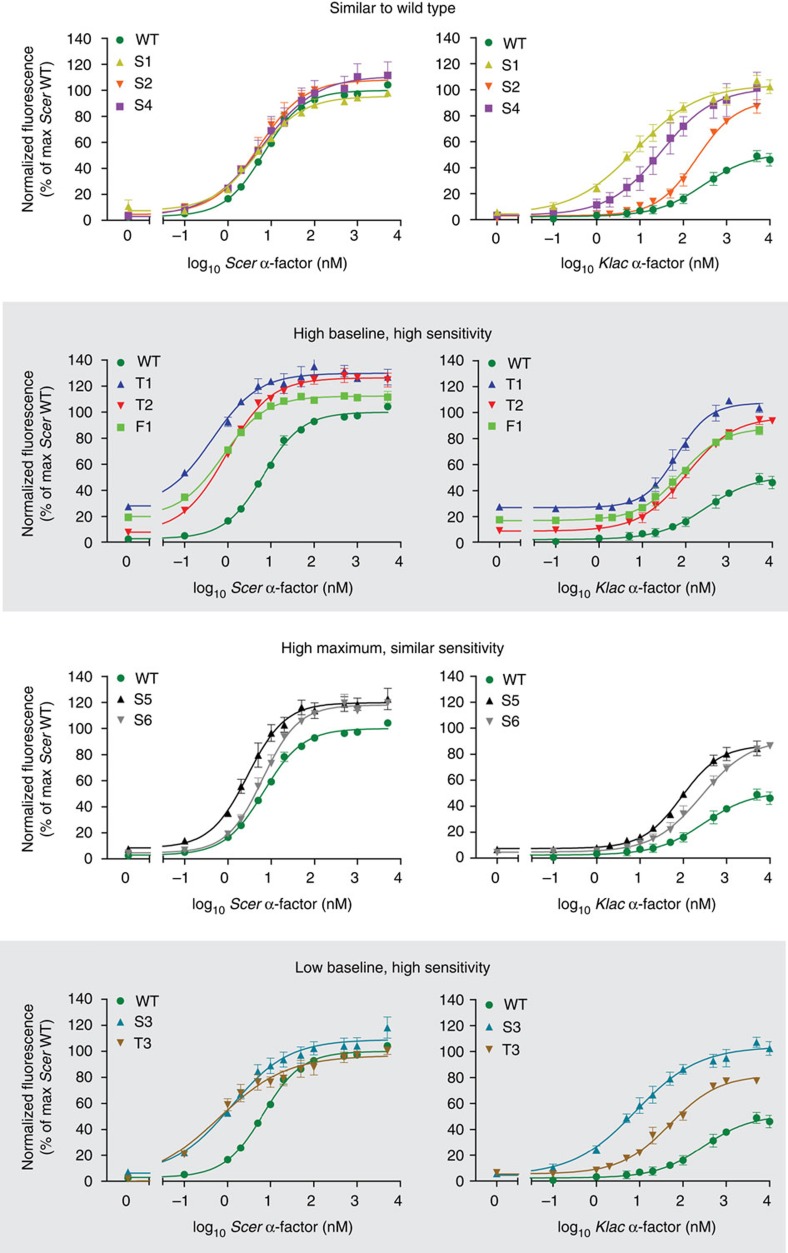
Ste2 variants selected for their ability to confer a strong response to a foreign pheromone exhibit diverse response profiles. Dose-response profiles of selected Ste2 variants using either the native or foreign pheromone. Variants were clustered according to the shape of their response to *Scer* α-factor. Error bars represent the s.e.m..

To characterize Ste2 mutants in detail and uncover potential changes in receptor–network interactions, we focused on a subset of 10 variants with sites mutated more than once and/or mutated sites known to affect Ste2 signalling such as V280 (ref. 35) or C-terminal lysines^28^ (Table 1). We first measured the dose–response relationship of each variant with either *Klac* or *Scer* α-factor to identify possible phenotypic clusters. As shown in Fig. 2 (left column), we grouped mutants into four clusters based on differences in their sensitivity (EC50), baseline response and maximum response. Interestingly, the patterns uncovered with *Scer* pheromone were not found with *Klac* α-factor, with the latter yielding more diverse dose-response relationships (Fig. 2, right column). This diversity was probably a consequence of our selection regime, wherein the single concentration of foreign pheromone used (5 μM) imposed no constraints on the strength of the response at other concentrations, making various sensitivities and Hill coefficients permissible.

### Binding affinity does not explain all acquired sensitivity

Many of the selected Ste2 variants harboured mutations in the extracellular loops of the receptors or in regions that were previously implicated in ligand binding^36^,^44^,^45^. To differentiate between variants that had acquired a greater binding affinity for the foreign pheromone and those that had not, we measured receptor–ligand affinity in live cells using a fluorescently labelled pheromone and flow cytometry. As the assay was done with live cells, we were able to simultaneously measure the amount of receptors at the cell surface for each variant (*B*_max_). Cells were kept on ice during measurements to minimize the effects of ligand-induced receptor internalization, ensuring that *B*_max_ values are ligand independent.

**Figure 3:**
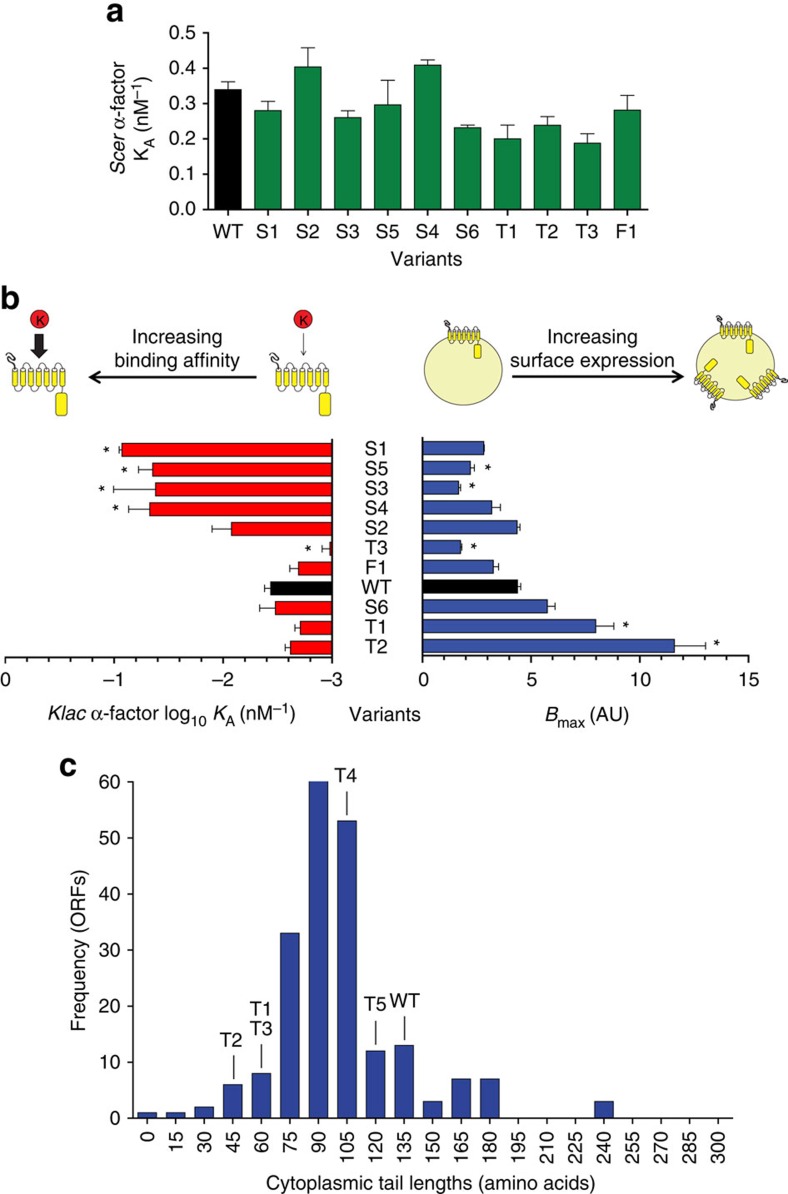
Ligand binding affinity and surface receptor expression suggest independent mechanisms behind sensitivity to *Klac* α-factor. (**a**) Binding constants of Ste2 variants for NBD-labelled *Scer* pheromone. Values were obtained from a saturation assay in live cells using flow cytometry. None of the Ste2 variants displayed a significant difference to wild-type (Dunnett's Multiple Comparison Test). Error bars represent the s.e.m. (**b**) Binding constants for *Klac* α-factor (left) plotted alongside surface receptor expression (right). Binding constants were obtained from a competition assay using a range of concentrations of *Klac* α-factor and a constant concentration of 20 nM NBD-labelled *Scer* α-factor. Surface expression was measured from a saturation assay. A subset of Ste2 variants exhibit high binding affinity for the foreign pheromone, while another subset displays high surface receptor expression. Asterisks indicate statistically significant differences to wild-type (*P*<0.05, Dunnett's Multiple Comparison Test). Error bars represent the s.e.m. (**c**) Fungal Ste2 homologues were obtained from PSI-BLAST and transmembrane domain topologies were predicted by TMHMM^48^. The lengths of C-terminal tails were extrapolated from the predicted topologies and plotted as a frequency distribution. This wide distribution encompasses the lengths of our Ste2 tail truncation mutants (indicated by labels above the bars).

We found that all variants tested displayed a strong binding affinity to *Scer* α-factor (Fig. 3a). Conversely, we observed important differences across variants when comparing *B*_max_ values and *Klac* α-factor affinity (Fig. 3b). Half of the variants assayed displayed a stronger affinity for *Klac* α-factor than the wild type (4 out of 10 when considering statistical significance), but had no increases in surface receptor expression. Of the remaining variants, three were highly expressed at the surface (two were significant). Interestingly, four variants had dramatic changes in the receptor's cytoplasmic tail resulting from either a premature stop codon or a frameshift mutation.

Among the mutant receptors showing a high binding affinity for *Klac* α-factor, variants S1, S2 and S4 showed an unchanged response sensitivity to *Scer* α-factor, but different sensitivities to *Klac* α-factor (Fig. 2). For these, the ligand-specific effects of their mutations suggested that changes at their binding site specifically contributed to their phenotype. However, the presence of several low-affinity variants led us to conclude that a stronger receptor–ligand interaction was not the sole evolutionary path favoured by our directed evolution experiment. For such variants, we had to consider other possible mechanisms.

### GPCR tail length variability is found in natural evolution

We were surprised by the prominence of C-terminal tail truncations among low-affinity variants due to the role of this region in numerous regulatory interactions and the apparent severity of this type of mutation. In total, we isolated five variants with premature stop codons with tail lengths of 39, 50, 58, 103 and 124 amino acids. This remarkable tolerance to truncations suggests that this region does not adopt a well defined structure^46^ and this was confirmed with FoldIndex^47^, a predictor of disordered regions (Supplementary Fig. 2). To determine whether cytoplasmic tail length variability is a natural trait of GPCRs, we examined the distribution of tail lengths in fungal Ste2 homologues. Through PSI-BLAST and the transmembrane domain predictor TMHMM^48^, we retrieved 225 fungal GPCR and their cytoplasmic tail sequences. We found a wide distribution of tail lengths, ranging from less than 15 amino acids to about 240 amino acids, which encompassed those of our selected Ste2 variants (Fig. 3c). This confirmed that the truncated receptors selected in our study resemble Ste2 homologues found in nature and pointed to numerous truncation-elongation events in the evolutionary history of GPCRs.

We hypothesized that the partial loss of the cytoplasmic tail seen in some variants was a driving mutation behind *Klac* α-factor sensitivity. Notably, variants T1 and T2 contained a premature stop codon in their C-terminal tail and only one other mutation occurring outside of this region. We found that removing the latter mutation, as well as the sequence downstream of the stop codon to eliminate any possible read-through did not affect the overall properties of the truncated receptors (now called T1* and T2*; Supplementary Table 2), suggesting that their phenotype could be attributed to the truncation. To determine how tail truncations affected the function of the evolved receptors, we subsequently focused on these mutants.

### Partial tail truncations impair the Ste2–Sst2 interaction

The receptor's C-terminal tail has been linked to two aspects of negative pathway regulation: receptor internalization (also called receptor endocytosis) and Sst2 recruitment. We confirmed via receptor–GFP fusions that T1* and T2* were defective at internalizing, both with and without pheromone (Supplementary Fig. 3). We reasoned that this was the cause of their high *B*_max_ values due to the accumulation of receptors at the cell surface. In principle, one might expect that higher surface receptor expression would enable the sensing of lower pheromone concentrations, resulting in a more sensitive response. However, past studies have shown that defective endocytosis has only a minor effect on sensitivity to *Scer* α-factor^49^ and this may only be apparent during long-term exposure to pheromone^50^. Likewise, impaired homo-oligomerization, a key feature of endocytosis, does not appear to greatly affect signalling although this has not been studied systematically^51^. Finally, the impact of receptor overexpression is more controversial^22^,^52^,^53^,^54^.

To understand these effects, we constructed a mathematical model of a simplified Ste2 system (Supplementary Fig. 4, Supplementary Note 1). Our model confirmed that impaired receptor endocytosis results in the accumulation of receptors at the cell surface, but this was not predicted to shift the sensitivity of the response. Instead, our model predicted that the EC50 could be altered by unequal changes in the basal and induced rates of receptor internalization. This result was also observed in an analogous model focusing on the epidermal growth factor receptor^55^. However, based on simulations with many experimentally derived parameter values, we found that Ste2 mutants solely defective in endocytosis would only show modest effects on sensitivity.

Our mathematical model led us to consider the receptor's interaction with the RGS Sst2 as the main source of the sensitivity shift. Ste2 is thought to bring Sst2 in proximity of its membrane-anchored target, the GTPase subunit of the heterotrimeric G protein, through a physical interaction that enables efficient pathway deactivation^22^. This interaction is abolished in the RGS mutant Sst2^Q304N^, and this confers a greater sensitivity to the native pheromone^56^.

**Figure 4:**
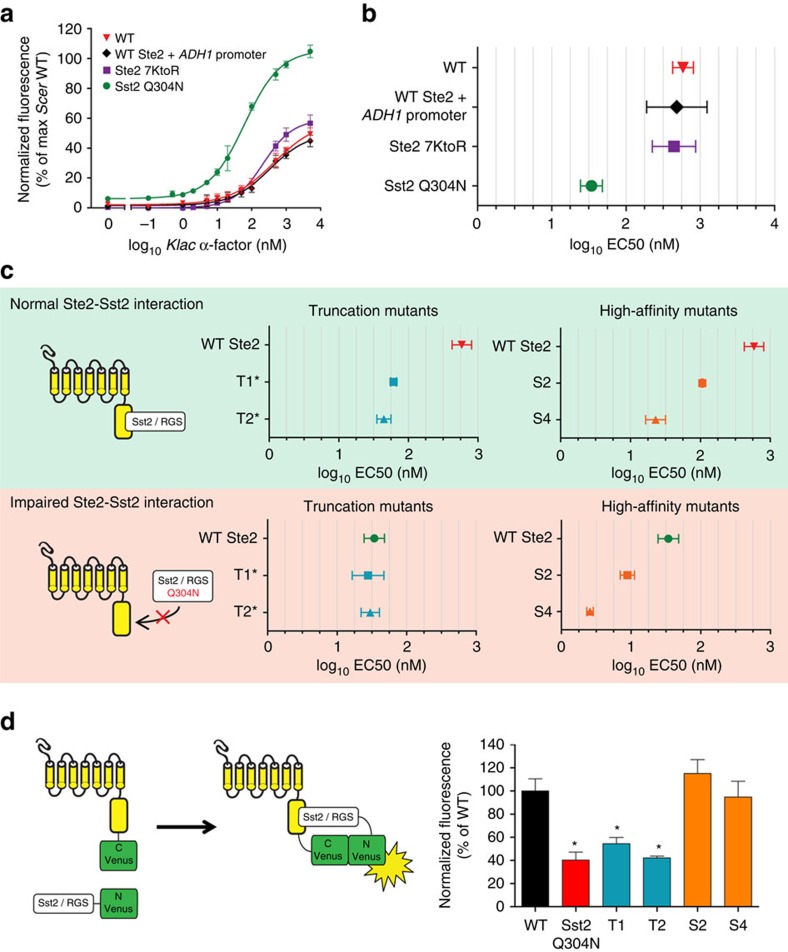
A decreased interaction with Sst2 explains the sensitivity shift of partially truncated receptors. (**a**) Dose-response relationship of various Ste2 or Sst2 constructs with *Klac* α-factor. Only the variant Sst2^Q304N^, which does not interact with the receptor, enables a stronger response to the foreign pheromone. (**b**) Mating response sensitivity for the Ste2 or Sst2 constructs assayed in **a**. EC50 values were derived from duplicate dose-response experiments. (**c**) Sensitivity of the mating response conferred by Ste2 variants alone or in combination with Sst2^Q304N^. The sensitivity was not additive for truncated receptors. (**d**) Bimolecular fluorescence complementation of Venus fragments fused to Ste2 and Sst2. Venus fluorescence was measured by flow cytometry in duplicate experiments and normalized by the *B*_max_ values of the Ste2-C-Venus variants. Venus fluorescence was lower when cells expressed Sst2^Q304N^ or truncated Ste2, suggesting that these mutations impair the interaction between the receptor and its RGS. Asterisks indicate statistically significant differences to wild-type (*P*<0.05, Dunnett's multiple comparison test). All error bars represent the s.e.m.

To experimentally test whether a greater sensitivity to *Klac* α-factor could result from receptor overexpression, impaired endocytosis and/or Sst2 recruitment, we measured *Klac* α-factor sensitivity under each scenario. First, we designed a gene construct in which wild-type receptor expression was driven by the strong promoter of the gene *ADH1* (p*ADH1*). The *B*_max_ value for p*ADH1*-expressed Ste2 was 6.05±0.65-fold higher than that of Ste2 expressed from its endogenous promoter. Second, we obtained a Ste2 variant in which all C-terminal lysines involved in endocytosis were substituted to arginine, dubbed 7KtoR^28^. This variant is defective in endocytosis but not in Sst2 recruitment^22^. Third, we co-expressed the wild-type receptor with Sst2^Q304N^. The resulting dose-response curves and their EC50 values are shown in Fig. 4a,b, respectively. Strikingly, we found that among the three scenarios, only Sst2^Q304N^ conferred improved sensitivity to *Klac* α-factor. Since we allow GFP expression to proceed for 3 h before making measurements, we considered the possibility that signalling under conditions of high receptor expression or low endocytosis was quashed by Sst2 over time. Therefore, we measured MAPK phosphorylation 30 min after *Klac* α-factor treatment by western blotting. Once again, only Sst2^Q304N^ showed high MAPK phosphorylation levels (Supplementary Fig. 5).

We proceeded to determine whether our truncated receptors were more sensitive to *Klac* α-factor due to an altered interaction with Sst2. We reasoned that if a partial tail truncation weakened the receptor's interaction with Sst2, its effect would be equivalent to that caused by the Q304N mutation in Sst2 and, in consequences, the two mutations combined should not display an additive phenotype. We co-expressed truncated receptors or high-affinity variants with Sst2^Q304N^ and measured cells' sensitivity to *Klac* pheromone. We found that the sensitivity conferred by Sst2^Q304N^ was not improved when receptor truncations were co-expressed (Fig. 4c). Conversely, the high binding affinity variants S2 and S4 produced an even more sensitive response when co-expressed with Sst2^Q304N^.

While these results pointed to an altered interaction between truncated receptors and Sst2, we sought to determine if the interaction was in fact physically weaker rather than simply improper and to what extent. For this, we used bimolecular fluorescence complementation with Venus fluorescent protein^57^,^58^. In this assay, Ste2 and Sst2 are fused to the C- and N-terminal halves of Venus, respectively. On physical interaction, Venus is reconstituted and its fluorescence can be detected by flow cytometry and normalized to protein expression levels (Supplementary Table 3). The Venus fluorescence for different construct combinations is presented in Fig. 4d. We found that wild-type Ste2 and Sst2 produced significantly more fluorescence per Ste2 molecule than wild-type Ste2 and Sst2^Q304N^, confirming the reduced physical interaction found by Ballon *et al*^22^. Remarkably, the truncated receptors T1 and T2 also caused lower Venus fluorescence than wild-type, though higher than Sst2^Q304N^. On the other hand, the two binding mutants S2 and S4 showed similar Venus fluorescence compared with wild-type. Together, these results suggest that the sensitivity of partial tail truncations to the foreign pheromone can be attributed to an impaired interaction with Sst2.

### Partial receptor truncations impair Sst2 activity

We further investigated the effects of receptor truncations on Sst2 activity and pathway regulation. In the absence of a Ste2–Sst2 interaction, either through a point mutation in the RGS or by truncating the entire C-terminal tail of the receptor, Sst2 is deficient in promoting signal downregulation and recovery from pheromone-induced cell cycle arrest^22^. However, it was unclear if partial receptor tail truncations, such as those found in T1* and T2*, would have similar consequences. Past work suggests that the receptor's third intracellular loop may also contribute to Sst2 function^33^, possibly as an additional contact point which could offset the effects of partial truncations.

An important feature of normal Sst2 function is fast pathway deactivation as reflected by the dephosphorylation of Fus3, the pathway's MAPK^56^. Since pathway activation causes cell cycle arrest, a rapid shut-off ensures that cells without a compatible mate are able to resume growth. We reasoned that if partial tail truncations affected Sst2 function, the dynamics of MAPK dephosphorylation, as well as the rates of growth recovery, would reveal this.

**Figure 5:**
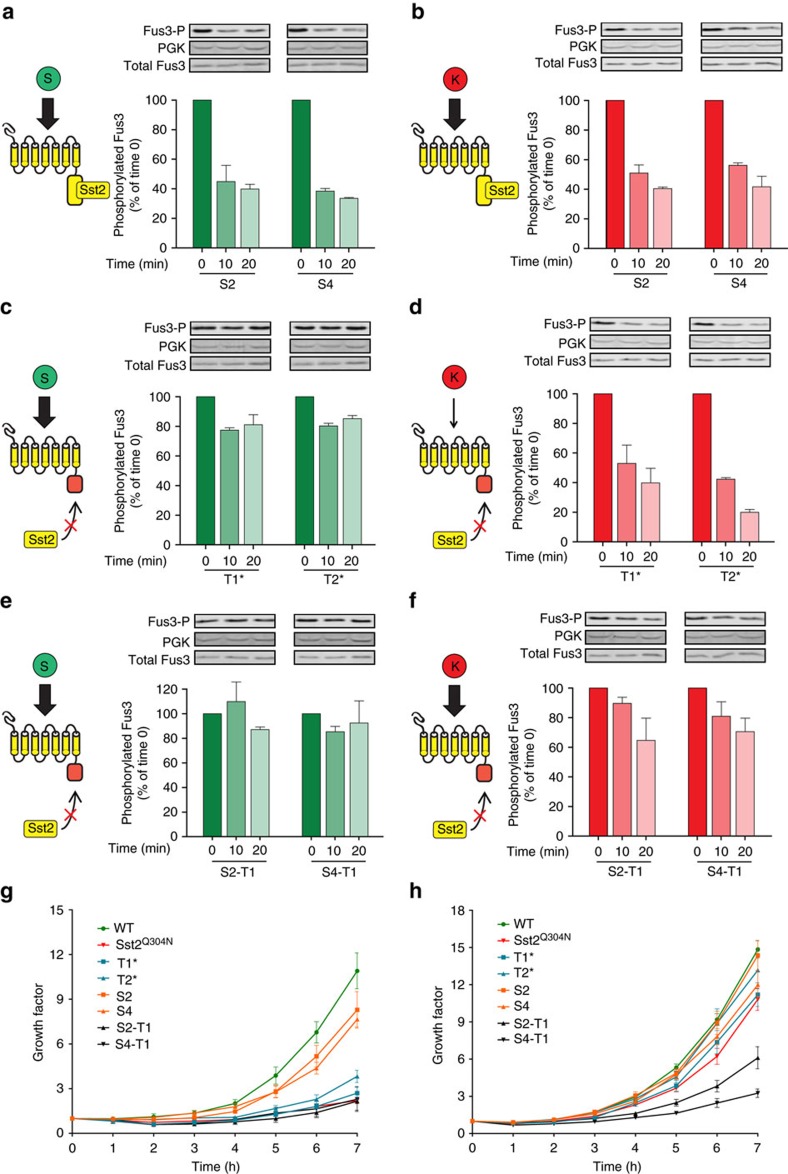
MAPK phosphorylation dynamics and growth curves reveal the role of the receptor-RGS interaction in controlling both mating pathway activation and deactivation. (**a**–**f**) Levels of phosphorylated Fus3 after pheromone wash-off for different Ste2 variants. Following mating pathway activation with 3 μM pheromone cells, were washed in pheromone-free medium, incubated for the indicated time and lysed. Lysates were used for Western Blotting. Phosphorylated Fus3 levels were normalized to PGK levels and plotted. Green bar graphs indicate *Scer* α-factor, red indicated *Klac* α-factor. (**g**–**h**) Growth curves of cells expressing different Ste2 or Sst2 variants. Following mating pathway activation with 3 μM pheromone, cells were washed in pheromone-free medium, incubated for the indicated time and culture density was measured by flow cytometry. Values were normalized to the initial cell concentration to yield a growth ratio. All error bars represent the s.e.m.

We measured the levels of phosphorylated Fus3 following pheromone wash-off, as was previously done by Dixit *et al*^56^. Importantly, as we sought to determine whether binding affinity influenced this process, we performed this assay under mild washing conditions. As previously reported, we confirmed that levels of phosphorylated Fus3 declined rapidly in wild-type cells (Supplementary Fig. 6A), while the expression of Sst2^Q304N^ resulted in slower pathway deactivation (Supplementary Fig. 6B). We also observed that rapid pathway deactivation was preserved for the high-affinity variants S2 and S4, whether *Scer* or *Klac* pheromones were used to activate the pathway (Fig. 5a,b). In contrast, pathway regulation was more complex for the truncated receptors T1* and T2*. When treated with *Scer* α-factor, these variants exhibited slow Fus3 dephosphorylation (Fig. 5c) as predicted, further indicating that their partial tail truncations impaired normal Sst2 function. However, this effect was not observed when *Klac* α-factor was used to activate the pathway (Fig. 5d). Remarkably, cells expressing Sst2^Q304N^ and treated with *Klac* α-factor showed a similar result (Supplementary Fig. 6C,D).

The different rates of pathway deactivation could not be explained by the initial levels of phosphorylated Fus3, as these were all comparable for each pheromone (Supplementary Fig. 6E,F), which deactivated rapidly in all cases. Instead, these results pointed to a pheromone-specific effect, whereby a partial Sst2 function could still downregulate Fus3 phosphorylation provided that the receptor was activated by a weak ligand. We reasoned that if a truncated receptor were activated more potently by *Klac* pheromone, its deactivation would be slower. To test this, we generated hybrid mutants by truncating the high binding affinity mutants S2 and S4 to the same extent as T1*, which we called S2–T1 and S4–T1. As expected, these hybrid mutants displayed slow Fus3 dephosphorylation even when *Klac* pheromone was used, although this effect was less pronounced than with *Scer* pheromone (Fig. 5e,f). Remarkably, these results were mirrored in growth assays where cell density was measured for 7 h following pheromone treatment and wash-off (Fig. 5g,h). Rapid dephosphorylation correlated with rapid growth, while slow dephosphorylation matched slow growth.

**Figure 6:**
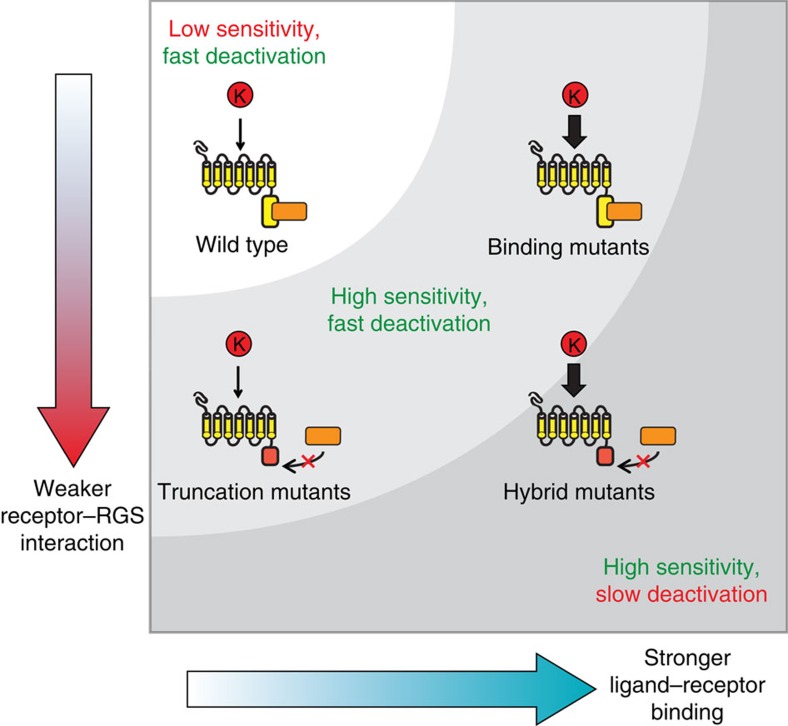
Pathway regulation depends on both ligand-receptor binding and a receptor-RGS interaction. Schematic map of the Ste2 evolutionary landscape. A stronger ligand-receptor interaction or a weaker receptor-RGS interaction can each promote mating response sensitivity to *Klac* pheromone, while a combination of the two is accompanied with slower pathway deactivation.

Taken together, these experiments suggest that Sst2 plays two distinct roles. First, Sst2 creates a threshold of receptor activation needed to activate the pathway, with partial Ste2 truncations enabling a response to a weak agonist by lowering this threshold. Second, Sst2 also contributes to pathway deactivation and this depends on both its interaction with the receptor and the affinity of the ligand (Fig. 6). Importantly, neither an altered Ste2–Sst2 interaction nor a high-affinity ligand alone can disrupt pathway deactivation. In the case of T1* and T2* treated with *Klac* α-factor, the interaction with Sst2 is impaired, thereby lowering the activation threshold that it provides, but with no consequences on deactivation, Sst2's second function. In this way, truncated receptors confer a strong response to *Klac* pheromone while preserving normal pathway deactivation and growth recovery.

### Mutants can mediate the formation of mating projections

An additional function of Ste2's C-terminal tail consists in regulating the proper formation of mating projections, also known as shmoos. Mating projections are a critical step in the fusion of haploid yeast cells, the end goal of mating pathway activation^18^. While full C-terminal truncations strongly affect shmoo orientation toward potential mates, the impact of partial truncations is less severe and they permit mating^59^,^60^. Recent work demonstrated that Sst2's RGS activity, but not its interaction with Ste2, is required for proper mating orientation^61^. To confirm this, we tested the ability of our truncated receptors to enable the formation of shmoos. Like their high-affinity counterparts, truncation mutants enabled the formation of typical mating projections based on morphology and frequency when treated with either pheromone (Supplementary Fig. 7). In contrast, only a few cells expressing wild-type Ste2 could shmoo when treated with *Klac* α-factor, and the shape of their mating projections was atypical. Together with GFP expression and MAPK phosphorylation, this data further confirms that mutant receptors confer a strong and biologically meaningful response to a foreign pheromone.

## Discussion

In this study, we investigated the role that mutations in a network hub can have in the evolution of a protein network. Our findings demonstrate that a GPCR can evolve the ability to respond to a weak agonist through at least two distinct mechanisms. The first is classical, and consists of an altered interaction between the receptor and the ligand reflected by an enhanced binding affinity. The second, which sheds light on an alternate evolutionary path, involves a change in the relationship between the receptor and its regulatory network. Specifically, we isolated multiple mutant Ste2 receptors with a truncated cytoplasmic tail, a region involved in protein–protein interactions. These truncated receptors exhibited various changes compared with wild-type Ste2, such as a higher surface expression and defective endocytosis, but their sensitivity to *Klac* α-factor was attributable to an impaired interaction with Sst2, a signal suppressor of G protein activity. Strikingly, these changes had a minimal impact on key aspects of pathway regulation. Our results thus demonstrate that it is possible to evolve a new receptor–ligand response by altering regulatory network interactions rather than the receptor's binding site. Furthermore, the prominence of truncated receptors in our selection, as well as the extensive cytoplasmic tail length variability that we observed in natural Ste2 homologues, suggest that evolutionary events that shorten or extend the cytoplasmic tail are rather common and may contribute to adaptive functional changes.

In a pioneering study performed over two decades ago, Lorraine Marsh used laboratory evolution to obtain Ste2 variants that could confer a mating response to the pheromone of *Saccharomyces kluyveri*^31^. In this way, she found various mutations affecting the receptor's ability to discriminate between the two ligands. However, due to a limited understanding of Ste2 signalling at the time, it was not possible to conclude on the precise molecular mechanisms behind the new phenotype. In the decades that followed, many more studies used mutagenesis and selection to characterize Ste2, often by focusing on a single region of the receptor and its hypothesized function. In this way, different Ste2 variants revealed the amino acid residues involved in pheromone binding^32^,^44^, signal transduction^32^,^37^,^38^, internalization^23^,^24^,^25^, oligomerization^30^,^62^ and signal regulation^22^,^29^,^33^,^62^. While these studies led to a greater understanding of the mechanisms behind the many facets of Ste2 signalling, their combined role in the evolution of new function remained unclear. Our work addresses this gap by shedding light on the molecular mechanisms through which a GPCR network can be linked to a foreign ligand.

As expected, our selection yielded several Ste2 variants with mutations affecting the receptor's binding site as revealed by their significantly enhanced binding affinity to *Klac* α-factor. Strikingly, all five high-affinity variants share the same three mutated sites: N216, V280 and S267. Mutations at the first two sites were previously shown to enable a pathway response to a weak agonist as well as an antagonist^34^,^35^. Both sites were also involved in suppressing a loss-of-function mutation at a known pheromone-binding residue, Y266 (ref. 34). Although S267 was not previously linked to pheromone binding, it is adjacent to Y266 and thus may also contribute to binding. The apparent broad specificity that arises when these residues are mutated, which we further confirmed with *Klac* and *Cgla* pheromones, initially suggested that such residues were ‘gatekeepers', ensuring the specificity of the Ste2-α-factor interaction. However, our results show that wild-type Ste2 binding is inherently promiscuous. Instead, the RGS Sst2 is required to impose a signalling threshold that is more stringent than binding specificity, allowing cells to discriminate between strong or weak ligands. Moreover, this threshold depends on a strong interaction between Sst2 and the receptor, as Sst2's presence alone is also insufficient to lock away the promiscuous potential of Ste2.

Despite the role of Ste2's cytoplasmic tail in signalling regulation, our data support the view that partially truncated receptors retain key aspects of pathway regulation. First, these variants did not result in constitutive signalling, indicating that overall pathway responsiveness was preserved. Second, the frequency and the morphology of mating projections in cells expressing truncated receptors were undistinguishable from those of wild-type cells, in agreement with a recent study that found that truncated variants can mediate mating^60^. Third, pathway deactivation proceeded normally in truncation mutants, provided that the receptor's interaction with the pheromone was weak. When cells expressed receptors combining a truncation with a binding affinity mutation, Fus3 dephosphorylation was slow with either pheromone. This suggests an interesting paradigm: if rapid pathway deactivation is to be preserved, then receptors can evolve either a stronger interaction with the ligand or a weaker interaction with Sst2, but not both. Since pathway activation causes cell cycle arrest, hybrid mutants may be disadvantaged unless they can mate efficiently, leading to additional selection pressure.

To conclude, our work highlights the prominent role of a receptor–network interaction in the evolution of a new ligand response. This scenario is of particular relevance in *S. cerevisiae* where interspecies mating has been a major evolutionary driver^63^, but its significance can be extended to other GPCR networks due to the underlying conservation that exists in this large family^64^,^65^. Through partial C-terminal truncations, the yeast GPCR Ste2 can acquire foreign pheromone sensitivity at little cost to signalling regulation. This has interesting implications in the study of GPCR evolution, where the highly variable length of the cytoplasmic tail has not been thoroughly examined. Our work suggests that this variability may not be random, but could instead be linked to network re-wiring events. Furthermore, the demonstrated importance of the RGS–tail interaction on response sensitivity and ligand discrimination may be relevant in GPCR-linked diseases. Alternative splice variants of GPCRs leading to truncated receptors are well documented, but little is known about their consequences beyond altered receptor trafficking^66^,^67^,^68^. If such splice variants are unable to interact with regulatory factors, particularly RGS proteins, hypersensitivity and a lack of ligand discrimination may play a role in their phenotype.

## Methods

### Mating pathway activation assays

Cells from three independent colonies were treated with one or a series of concentrations of pheromone and placed in a 30 °C shaking incubator. After 3 h, protein synthesis was inhibited by treating cells with 10 μg ml^−1^ cycloheximide. The intensity of the fluorescence in the 525/50 nm range was measured by analytical flow cytometry with a MACSQuant VYB (Miltenyi Biotec) with a 588 nm laser. Cells without receptors were used to subtract basal cell fluorescence from other samples to reveal the net GFP fluorescence. For dose-response assays, the data were fitted with the ‘log(agonist) versus response—Variable slope (four parameters)' model in Prism (GraphPad). All experiments included a wild-type Ste2 control. The fluorescence intensity was normalized to the maximum intensity of the wild-type control and multiplied by 100. EC50 values represent the mean of two experiments normalized to the wild-type value of each experiment.

### Mutagenesis and selection

For mutagenesis, the full-length wild-type *STE2* open reading frame (ORF) was amplified from pRS-*STE2* by error-prone PCR using the GeneMorph II kit (Agilent). PCR conditions (500 ng of template DNA, 20 cycles) were selected to yield a mean mutation rate of 4.0 DNA mutations per ORF. The resulting amplimers were ligated in pRS-p*STE2* and amplified in *E. coli* DH5α to generate a plasmid library of ∼50,000 Ste2 mutants. The library was transformed in the yeast strain RB001, yielding 20,000 colonies. For selection, yeast cells were treated with 5 μM *Klac* and incubated for 3 h in a 30 °C shaker. Cell sorting was done in a FACSAria (BD) while gating for high GFP fluorescence. In the first round, 19,000 events were sorted. In the second round, which was done using the cells recovered from the first round, 11,000 events were sorted. A total of 282 colonies recovered from both rounds were screened for their ability to activate the mating pathway better than wild type. Mutant *STE2* genes were extracted from the most promising colonies, transformed in naive cells to confirm that they conferred the phenotype, and sequenced.

### Pheromone-binding assays

To measure binding affinities and binding site levels, we used the NBD-labelled pheromone binding assay^69^. Cells from two independent colonies were treated with either varying concentrations of NBD-labelled *Scer* (saturation binding assays) or varying concentrations of *Klac* pheromone mixed with 20 mM NBD-*Scer* (competition-binding assays) and left on ice for 10 min with occasional mixing. Samples were processed by flow cytometry. For saturation binding, the data were fitted to the ‘One site—Total and nonspecific binding' model in Prism (GraphPad), and cells without receptors were used to account for nonspecific binding. This model uses the equation *Y*=*B*_max_[*L*]/(*K*_d_+[*L*])+*N*[*L*]+Background, where *Y* is the mean fluorescence, [*L*] is the concentration of labelled ligand and *N* is a proportionality constant for nonspecific binding. For competition binding, the background-subtracted data were fitted to the ‘One site—Fit Ki' model with the appropriate constraints. This model uses the equation *K*_i_=*IC*_50_/(1+[*L*]/*K*_D_). All experiments included a wild-type Ste2 control. *K*_D_, *B*_max_ and *K*_i_ values represent the mean of two experiments normalized to the wild-type values of each experiment.

### Predicting the lengths of C-terminal tails in Ste2 homologues

To identify the predicted disordered region, the amino acid sequence of *Scer* Ste2 was queried in FoldIndex^47^. PSI-BLAST for three iterations and with default parameters^70^. We restricted the search to matches of fungal origin, excluded *Scer* matches and discarded partial sequences. We submitted the resulting dataset to the transmembrane domain predictors TMHMM^48^ and extracted the predicted topology of sequences with seven transmembrane domains. As TMHMM also predicts the orientation of the helices, we discarded sequences for which the C-terminal tail was extracellular. From the resulting topologies, we measured the length of the cytosolic tail by defining its N-terminal boundary as the last amino acid of the seventh transmembrane domain, and its C-terminal boundary as the stop codon. The frequency distribution of the resulting amino acid lengths was plotted in Prism (Graphpad).

### Bimolecular fluorescence complementation

To measure the relative strength of the physical interaction between Ste2 variants and Sst2 variants, we used bimolecular fluorescence complementation with Venus fluorescent protein^57^,^58^. N- and C-terminal Venus fragments were generated by dividing the protein at amino acids 172–173. C-Venus was fused to Ste2 variants and N-Venus to Sst2 variants. Venus fluorescence was measured by flow cytometry as for GFP and normalized to the *B*_max_ of each receptor construct to account for receptor expression levels. *B*_max_ values were obtained with NBD-*Scer* as described above.

### Western blotting of MAPK deactivation

Activation and deactivation of the mating pathway was performed as described in ref. 56. Briefly, cells were treated with 3 μM pheromone and incubated in a 30 °C shaker for 30 min. The pheromone was washed off with fresh growth medium and cell aliquots were taken immediately after wash-off or following 10 and 20 min incubations. Cell lysates were resolved in a 10% SDS–PAGE gel and transferred to a PVDF membrane using a BioRad Trans-Blot Turbo. Membranes were blocked overnight with Licor Odyssey TBS-formulated blocking buffer. Blotting of Fus3-P was done with a primary anti-p44/42 MAPK antibody (Cell Signaling Technology, #4370) diluted by 1:2,000, followed by the secondary antibody IRDYE 800 (Licor, #926–32211). Blotting of total Fus3 was done with a primary anti-Fus3 antibody (Santa Cruz Biotechnology, #sc6773) diluted by 1:5,000, followed by the secondary antibody IRDYE 800 (Licor, #926–32214). Blotting of the loading control phosphoglycerate kinase (PGK) was done with a primary anti-PGK antibody (Invitrogen, #459250) diluted by 1:5,000, followed by the secondary antibody IRDYE 680LT (Licor, #926–68020). All secondary antibodies were diluted by 1:10,000. Bands were visualized with a Licor Odyssey CLx infrared imaging system (Licor). Band intensity was extracted with Image Studio Lite (Licor). The intensities of Fus3-P bands were normalized to that of the PGK loading control. The experiment was performed in duplicates.

### Cell growth recovery assay

To measure recovery from cell cycle arrest, cells were first treated with 500 nM pheromone and incubated in a 30 °C shaker for 30 min. The pheromone was then washed off with fresh growth medium. Culture aliquots were taken immediately after wash-off (time zero) and subsequently every hour for 7 h. Aliquots were used to measure culture density by flow cytometry. Culture densities were normalized to time zero to obtain growth factors.

### Data availability

The data that support the findings of this study are available from the corresponding author on request. In addition, the uncropped versions of the western blots featured in this article are available in Supplementary Figs 8–10.

## Additional information

**How to cite this article:** Di Roberto, R.B. *et al*. Evolution of a G protein-coupled receptor response by mutations in regulatory network interactions. *Nat. Commun.* 7:12344 doi: 10.1038/ncomms12344 (2016).

## Supplementary Material

Supplementary Information Supplementary Figures 1-10, Supplementary Tables 1-8, Supplementary Note 1, Supplementary Methods and Supplementary References

## Figures and Tables

**Table 1:** Ste2 variants and their dose-response sensitivity to either pheromone.

**Name**	**Protein mutations**	***Scer*** **EC50 (nM)**	***Klac*** **EC50 (nM)**
WT	NA	8.74±2.2	726±240
S1	I82N, N216D, Y266F	4.36±0.42	19.1±6.3
S2	V280I	5.90±1.2	107±2.5
S3	S267C,V280D, K358R,T414M	1.73±0.096	6.24±1.4
S4	N216S	8.45±0.11	24.1±7.6
S5	S267R, T282S	3.30±0.19	86.6±28
S6	M54V, A62T, M69L, G115R	7.81±0.11	1500±620
T1	Y30H, K358*	0.375±0.14	80.4±12
T2	T78M, A336D, K337P, S338E, S339*	0.909±0.20	100±8.9
T3	G237D, F312L, R350*	0.758±0.0049	84.7±20
F1	N25D, K202T, T309N, A397E, Fs401	0.649±0.27	224±1.8

NA, not applicable; WT, wild type.

Asterisks designate a premature stop codon while ‘Fs' designates a frameshift mutation.

Values represent the mean±s.e.m.
