# Supplementary material for: Evolution of a G protein-coupled receptor response by mutations in regulatory network interactions
Source: Nat Commun. 2016 Aug 4;7:12344. doi: 10.1038/ncomms12344 (PMC4976203; doi:10.1038/ncomms12344)
Supplement: Supplementary Information — Supplementary Figures 1-10, Supplementary Tables 1-8, Supplementary Note 1, Supplementary Methods and Supplementary References [file ncomms12344-s1.pdf]

SUPPLEMENTARY INFORMATION

SUPPLEMENTARY FIGURES

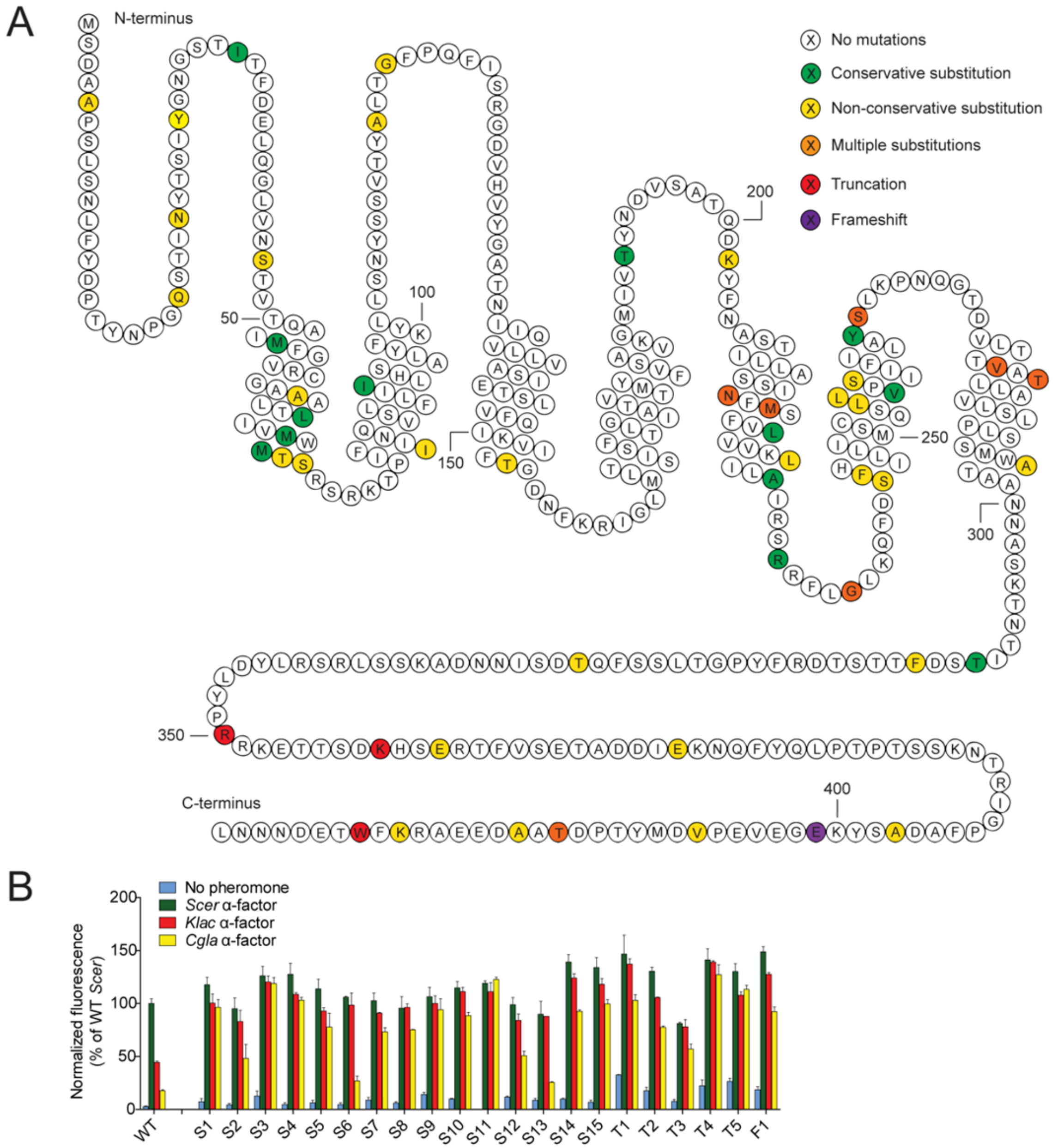

Supplementary Figure 1: The Ste2 variants selected show a diversity of mutations, a robust native activity and a concomitant increase in promiscuity. A, Snake plot of the G protein-coupled receptor Ste2. Mutated amino acid residues are highlighted according

to the legend in the top right. **B,** GFP fluorescence was measured in cells treated with 5 μM pheromone, incubated for 3 hours and analyzed by flow cytometry. All variants retained their ability to response to *Scer* α-factor, and many variants acquired the ability to respond to *Cgla* α-factor, which was not a criterion for selection. Error bars represent the s.e.m.

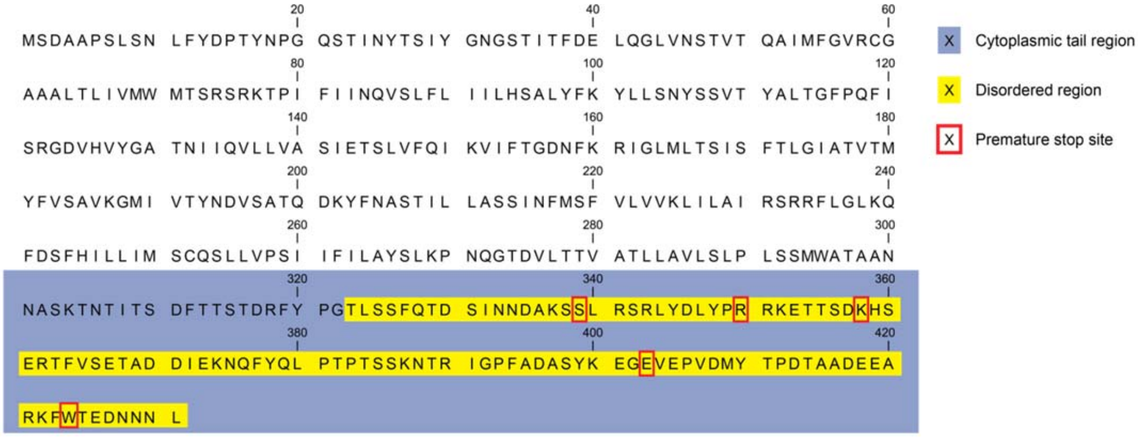

Supplementary Figure 2: The Ste2 cytoplasmic tail is disordered. The predicted disordered region of Ste2 was obtained from FoldIndex© [9]. The blue-shaded area indicates the cytoplasmic tail region, amino acids highlighted in yellow are predicted to be unfolded and boxed residues indicate the premature STOP sites of our truncation mutants. The predicted disordered region encompasses most of the cytoplasmic tail.

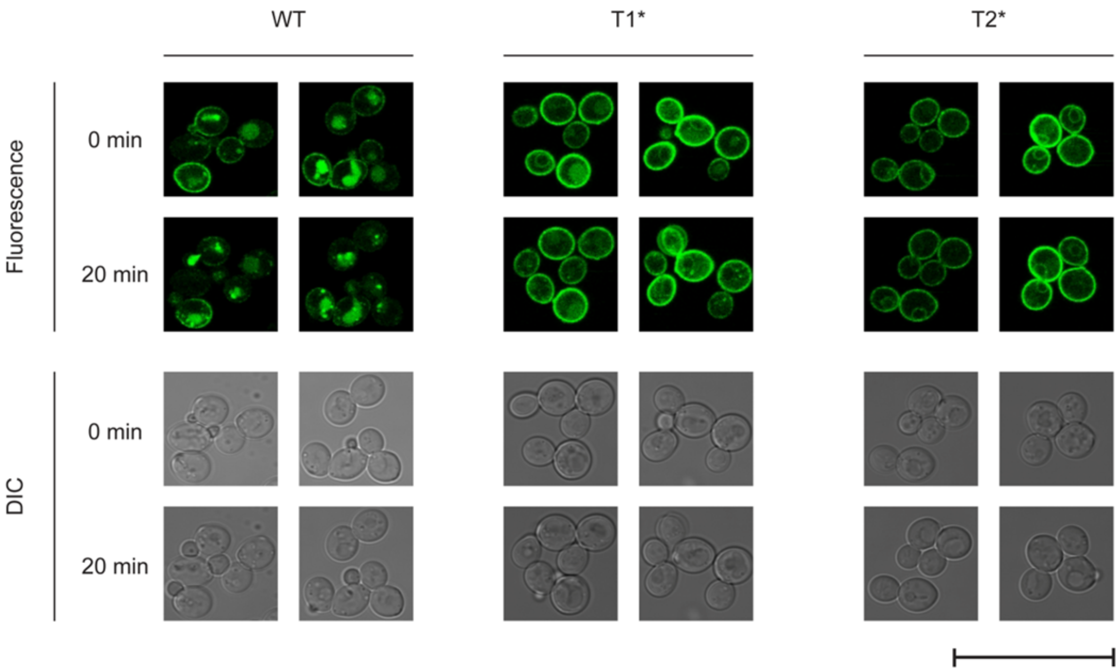

Supplementary Figure 3: Truncated receptors show defective internalization. Cells expressing Ste2-GFP fusions were visualized by fluorescence microscopy before and after treatment with 5 μM *Scer* pheromone. The top two rows show fluorescence and the bottom two rows show differential interference contrast (DIC). While the WT receptor internalizes upon addition of pheromone (GFP signal moves from the plasma membrane to endosomes), the GFP signal of the truncated receptors remains largely at the plasma membrane after pheromone induction. Scale bar in bottom right represents 25 μm.

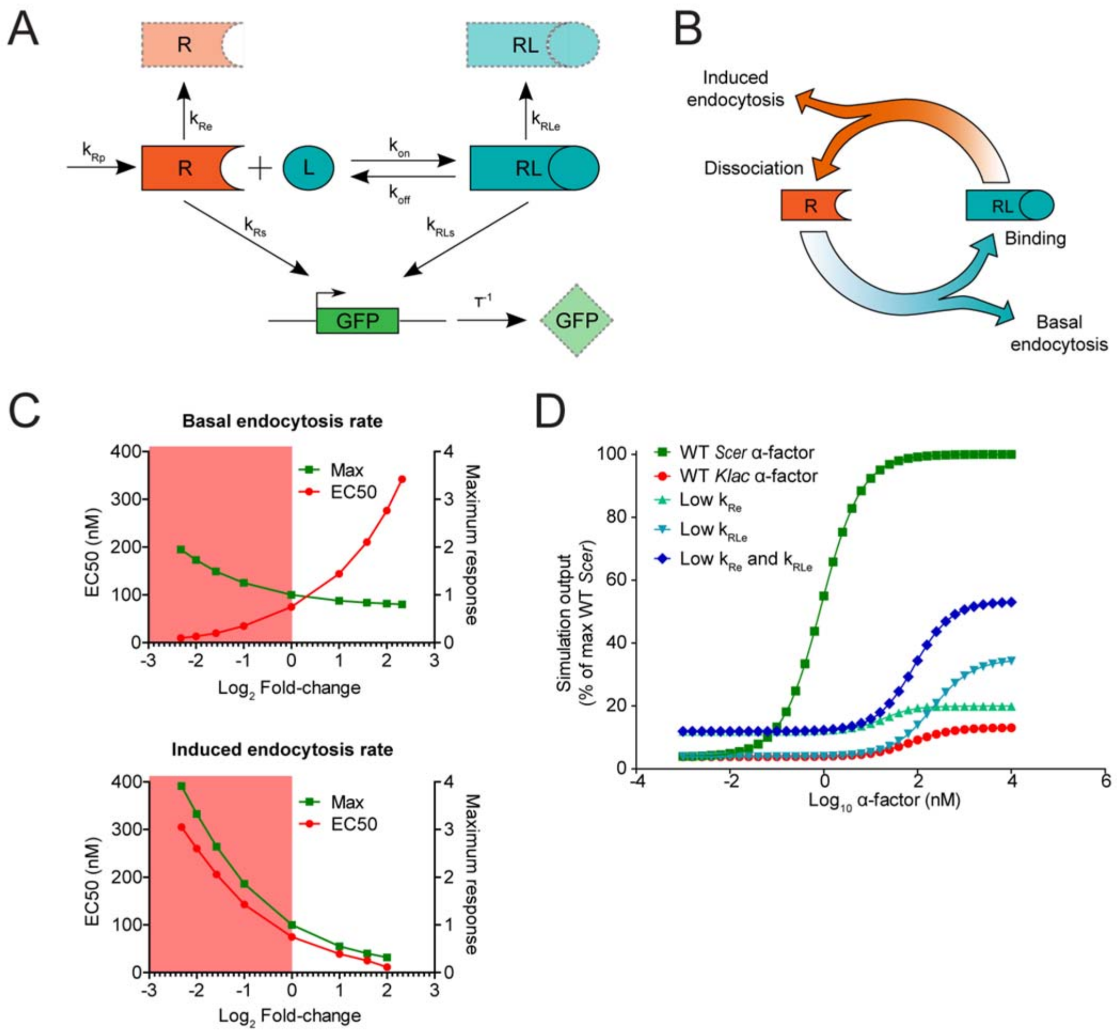

Supplementary Figure 4: A simplified model of the Ste2 signaling network explains the mixed effects of receptor endocytosis on pathway sensitivity. A, Free Ste2 receptors (R) bind to their cognate pheromone ligand (L), forming a receptor-ligand complex (RL). Both are internalized and degraded (dotted proteins). Only non-internalized receptors can signal by promoting GFP expression from a mating-responsive promoter. GFP is degraded at a basal rate. Reactions and their rate constants are indicated by labeled arrows. **B**, Conceptual view of the bound-unbound receptor equilibrium. The equilibrium is dictated by both binding and endocytosis kinetics. **C**, Theoretical effects of altering endocytosis rates on sensitivity and maximum response. Fold-changes are

applied on wild-type values. Shaded red area represents lower rates. **D**, Simulated dose-response relationships with experimentally-derived endocytosis rates. Lower rates of endocytosis do not greatly improve the response to *Klac* pheromone without losing sensitivity.

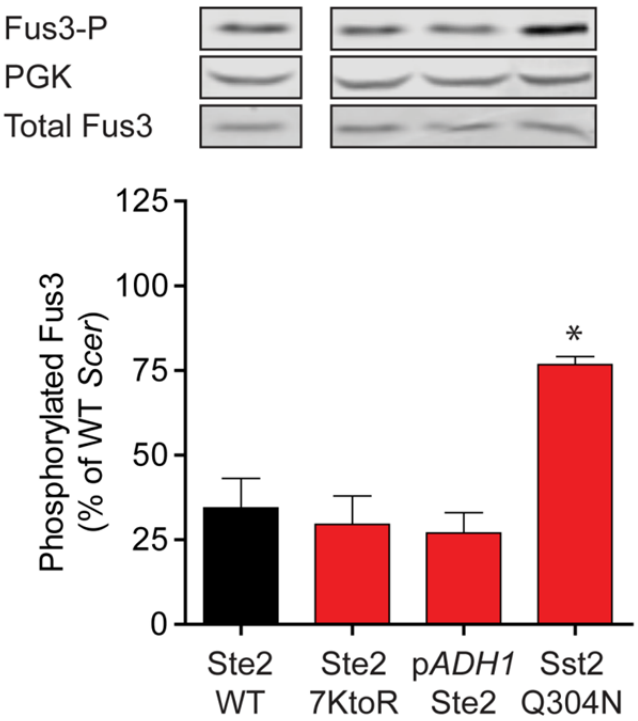

Supplementary Figure 5: MAPK phosphorylation confirms the ability of Sst2^{Q304N} to enable a strong response to *Klac* α-factor with WT Ste2. Pathway activation was measured in cells treated with 3 μM pheromone, incubated for 30 min and lysed for Western Blotting. Phosphorylated Fus3 levels were normalized to PGK levels and plotted. The asterisk indicates a statistically significant difference to wild-type ($P < 0.05$, Bonferroni-Corrected *t*-test). Error bars represent the s.e.m.

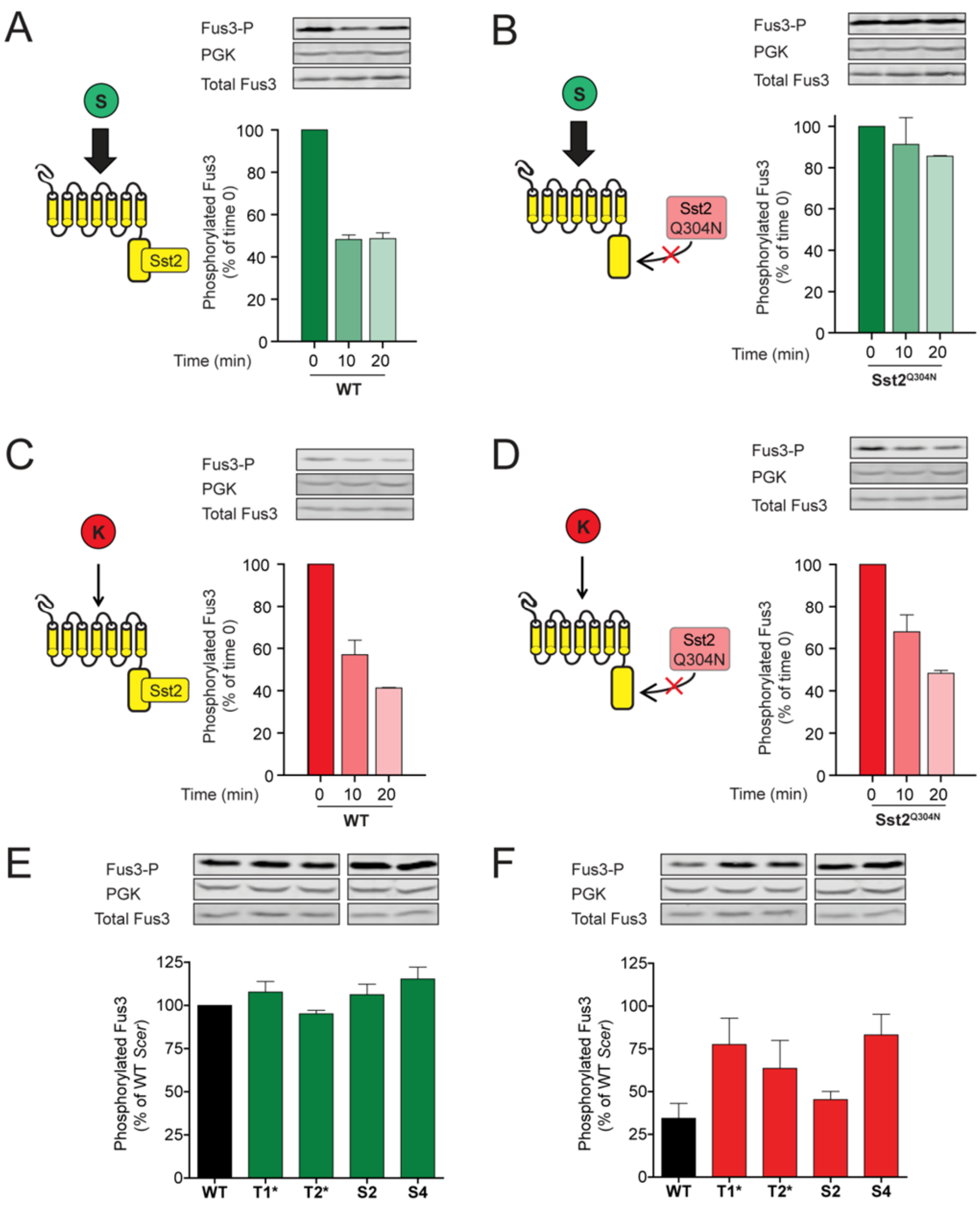

Supplementary Figure 6: Cells expressing wild-type Sst2 and Sst2^{Q304N} show different rates of Fus3 de-phosphorylation. A-D, Levels of phosphorylated Fus3 following pathway deactivation, normalized to time zero. Pheromone-treated cells were washed once, resuspended in pheromone-free medium, incubated for the indicated time and lysed. **E-F,** Levels of phosphorylated Fus3 following pathway activation with either

Scer α-factor (A) or *Klac* α-factor. Cells were treated with 3 μM pheromone, incubated for 30 min and lysed. Lysates were used for Western Blotting. Phosphorylated Fus3 levels were normalized to PGK levels and plotted. Green bar graphs indicate *Scer* α-factor, red indicated *Klac* α-factor. Variants assayed are indicated in bold. Error bars represent the s.e.m.

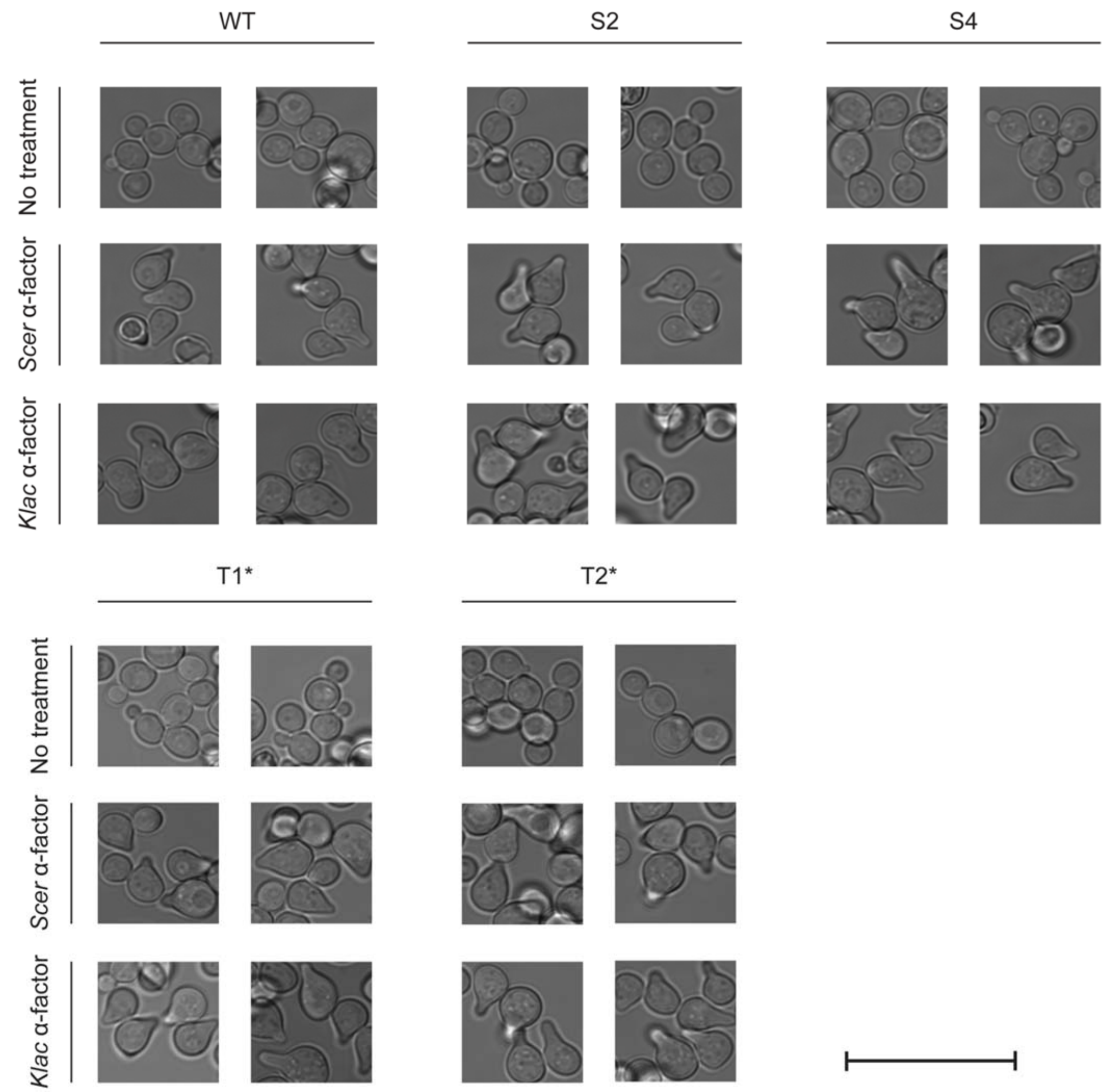

Supplementary Figure 7: Mating projections of cells expressing Ste2 variants are typical with both pheromones. Mating-competent cells expressing Ste2 variants were visualized by differential interference contrast microscopy before and after treatment with 5 μM pheromone. Cells expressing wild-type receptor form normal mating projections in response to *Scer* α-factor, while *Klac* α-factor induces abnormal shmoo formation. Cells expressing mutant receptors shmoo normally with either pheromone. We observed no shmoo in the absence of pheromone for any variant. Scale bar in bottom right represents 25 μm.

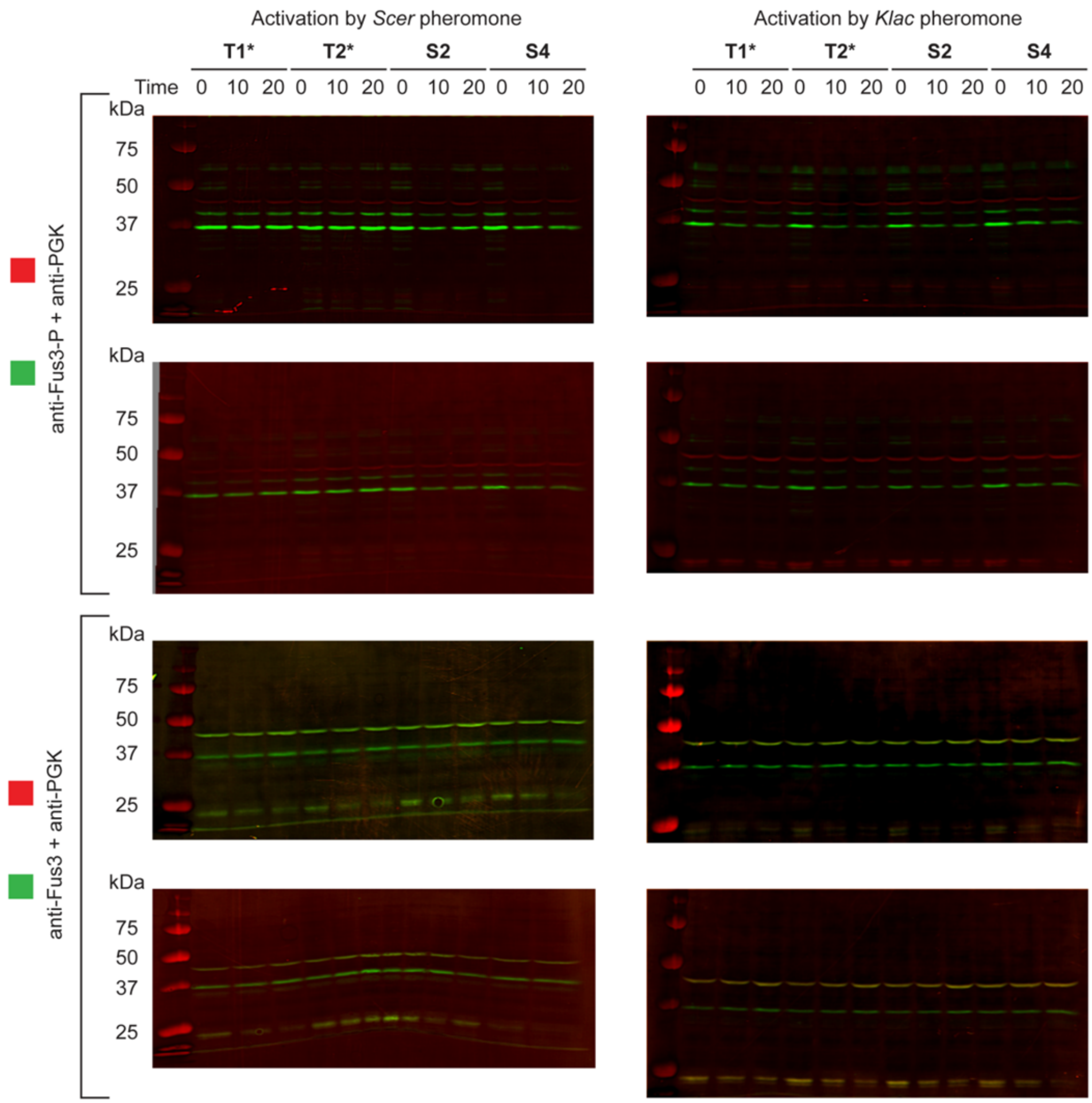

Supplementary Figure 8: Full Western blots of Figure 5 (mating pathway deactivation). Uncropped Western blots of mating pathway deactivation featured in Figure 5 using *Scer* (left blots) or *Klac* pheromone (right blots). Green bands represent phosphorylated Fus3 (top four blots) or total Fus3 (bottom four) while red bands represent either the loading control PGK or the molecular weight marker bands.

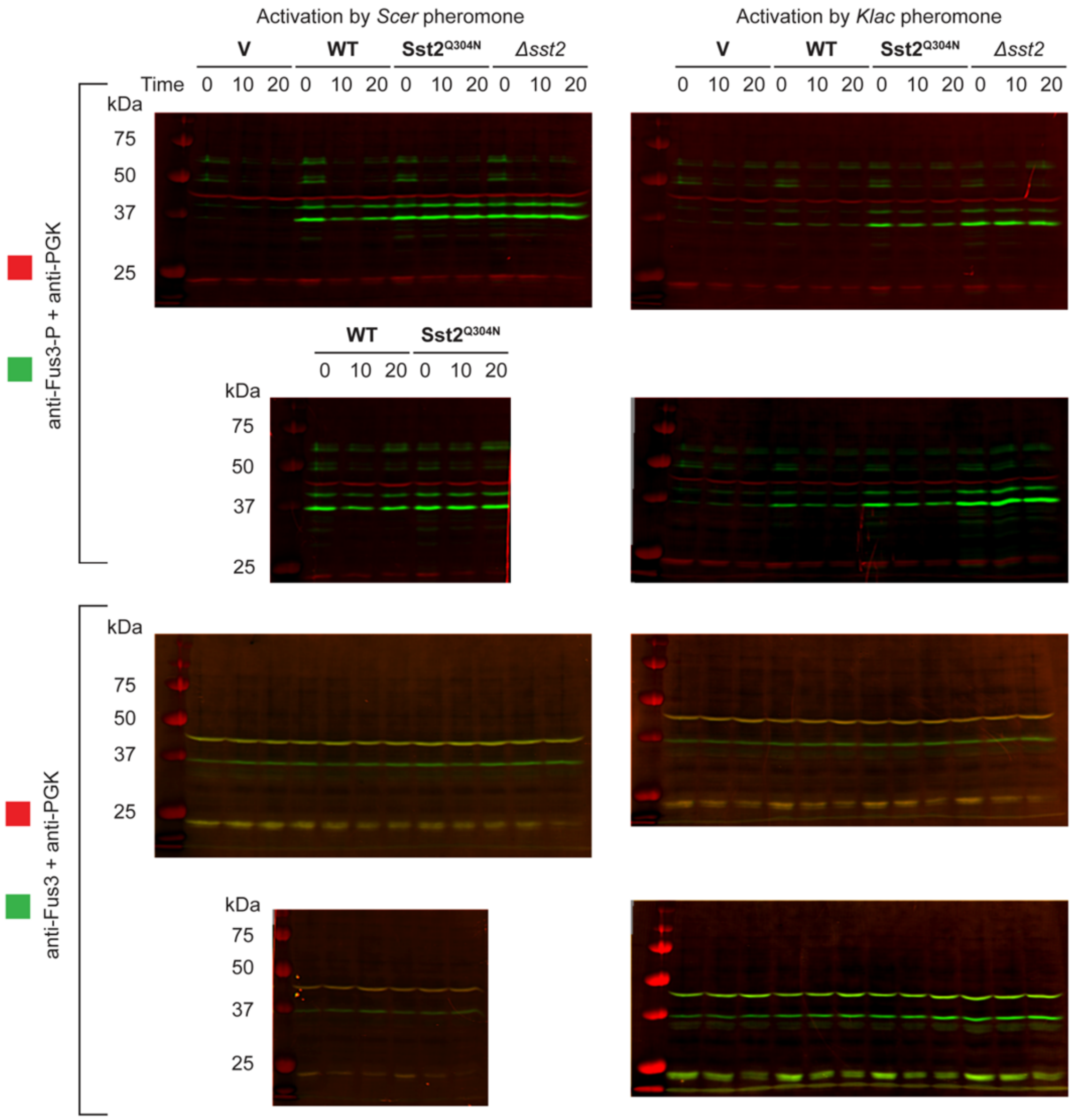

Supplementary Figure 9: Full Western blots of Supplementary Figure 6 (mating pathway deactivation). Uncropped Western blots of mating pathway deactivation featured in Supplementary Figure 6 using *Scer* (left blots) or *Klac* pheromone (right blots). Green bands represent phosphorylated Fus3 (top four blots) or total Fus3 (bottom four) while red bands represent either the loading control PGK or the molecular weight marker bands.

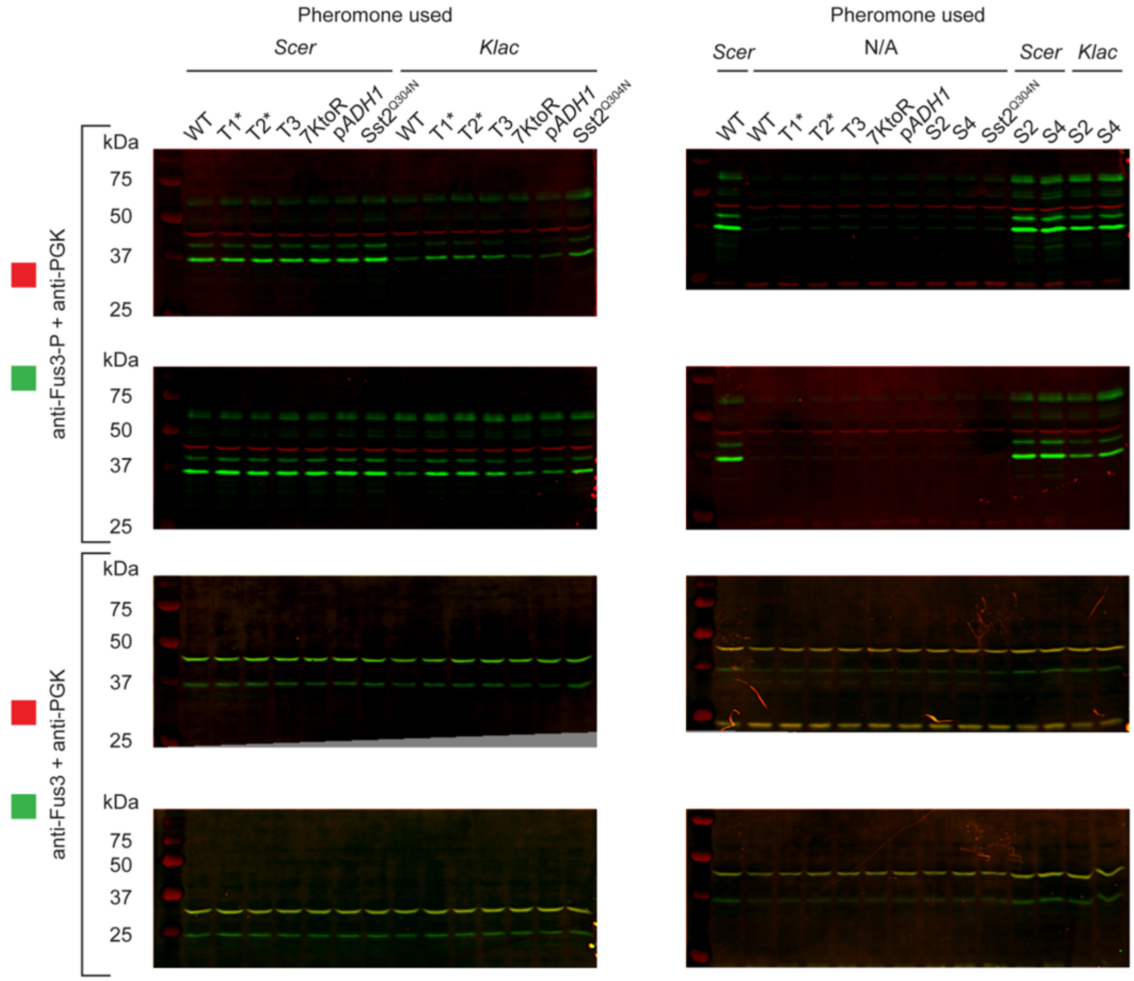

Supplementary Figure 10: Full Western blots of Supplementary Figures 5 and 6 (initial mating pathway activation). Uncropped Western blots of initial mating pathway activation featured in Supplementary Figures 5 and 6 using *Scer* or *Klac* pheromone. Green bands represent phosphorylated Fus3 (top four blots) or total Fus3 (bottom four) while red bands represent either the loading control PGK or the molecular weight marker bands.

SUPPLEMENTARY TABLES

Supplementary Table 1: Ste2 variants isolated following directed evolution.

Name	Selection round	Mutations
**S1**	1	I82N, **N216D**, **Y266F**
**S2**	1	**V280I**
**S3**	2	S267C, **V280D**, **K358R**, **T414M**
**S4**	2	**N216S**
**S5**	2	S267R, **T282S**
**S6**	2	**M54V**, A62T, M69L, G115R
**S7**	0[a]	I92M, **R233K**, F244L, **T414M**
**S8**	0[a]	T155A, **M218V**, **L255F**
**S9**	0[a]	**A229V**, E373V, A416T
**S10**	2	S73L, **T282A**, E361K
**S11**	2	L66M, A112T, L256S, **S259P**
**S12**	1	T192N, **G237S**
**S13**	1	I36V, S243C, L256M, **G273E**, A296T, Y345C, R350G, N388S
**S14**	2	A5T, **S47R**, L222M, V407D, **K422Q**
**S15**	2	Q21H, **L226F**, V257L
**T1**	1	**Y30H**, **K358***
**T2**	1	T78M, A336D, **K337P**, **S338E**, **S339***
**T3**	2	**G237D**, F312L, **R350***
**T4**	2	**I142N**, L146M, T179A, V191A, I308T, **K352M**, D370E, F394S, **E403***
**T5**	2	**M218I**, A296G, **T329I**, **W424***
**F1**	2	N25D, K202T, **T309N**, A397E, **Fs401**

[a]: Mutants isolated prior to FACS rounds. These were obtained from a random selection of unsorted mutants followed by a screen for mating pathway activation in the presence of *Klac* pheromone.

Mutations in **bold** affect sites previously implicated in Ste2 function.

Supplementary Table 2: Artificial truncation mutants and their properties

Mutant	T1	T1*	T2	T2*
***Scer* EC50**	0.375 ± 0.14	0.174 ± 0.026	0.909 ± 0.20	0.751 ± 0.071
***Klac* EC50**	80.4 ± 12	45.7 ± 11	100 ± 8.9	61.6 ± 5.7
Scer K_D	5.20 ± 1.0	5.19 ± 0.85	4.23 ± 0.43	5.84 ± 1.4
Klac K_i	517 ± 58	418 ± 9	420 ± 49	480 ± 100
B_{max}	7.97 ± 0.83	13.2 ± 1.3	11.6 ± 1.4	16.8 ± 3.3

Values represent the mean ± the standard error of the mean (s.e.m.)

Supplementary Table 3: B_{max} values of Ste2-C-Venus variants

Ste2 variant	Mean B_{max} ± s.e.m.
**WT**	1 ± 0.26
**T1**	3.19 ± 1.0
**T2**	4.52 ± 2.2
**S2**	0.738 ± 0.10
**S4**	0.805 ± 0.25

Supplementary Table 4: Model parameters and numerical values used in fits and simulations

Parameter	Wild-type value	Source	Alternative value	Source
k_{Rp}	3.12 nM min^{-1}	[4, 5, 6], our binding data[a]	N/A	
k_{on}	0.19 nM^{-1} min^{-1}	[7], our binding data[b]	0.0022 nM^{-1} min^{-1} (*Klac* pheromone)	Binding assays[b]
k_{off}	0.6 min^{-1}	[7]	N/A	
k_{Re}	0.0156 min^{-1}	[4]	0.005 min^{-1}	Binding assays[c]
k_{RLe}	0.0693 min^{-1}	[4]	0.023 min^{-1}	Binding assays[d]
k_{Rs}	$9.05 \cdot 10^{-6}$ min^{-1}	Median fit to dose-response[e]	N/A	
k_{RLs}	$1.42 \cdot 10^{-4}$ min^{-1}	Median fit to dose-response[e]	$1.70 \cdot 10^{-5}$ min^{-1} (*Klac* pheromone)	Median fit to dose-response[e]
τ	148 min	[8]	N/A	

[a]: Wild-type cells harbor 4900 STE2 molecules, corresponding roughly to a concentration of 200 nM for a cellular volume of 42 fL. The synthesis rate is calculated from equation (9), B_{max} values from our binding assays and the wild-type value of k_{Re}.
[b]: The binding rate is calculated using the k_{off} of Yi *et al.* and our measured K_D values.
[c]: The basal endocytosis rate is calculated using B_{max} values from our binding assays and the wild-type value of k_{Rp} which is assumed to be constant.
[d]: The alternative induced endocytosis rate is calculated by preserving the ratio between basal and induced rates.
[e]: The signaling ability of a receptor-ligand pair was obtained by fitting Equation (9) to dose response data.

Supplementary Table 5: Yeast strains used in this study

Name	Genotype	Origin
**SΦ992**	*W303 MATa, STE2, SST2, BAR1, FAR1, MFA2, his3, trp1, leu2, ura3*	[8]
**CB008**	*SΦ992 bar1::NatR, far1Δ*	[8]
**CB009**	*CB008 mfa2::pFUS1-GFP*	[8]
**RB001**	*CB009 ste2::TRP1*	This study
**RB002**	*CB008 ste2::TRP1*	This study
**RB003**	*RB001 sst2::URA3*	This study
**RB004**	*RB002 sst2::URA3*	This study
**RB005**	*SΦ992 ste2::TRP1*	This study
**RB006**	*RB005 sst2::URA3*	This study

Supplementary Table 6: Oligonucleotides used in this study

**Name**	**Sequence**	**Purpose**
Ste2KO_Trp3	AGGTGGTTTCTACCACTACTTACGAGATGTTTATTATGTAAAGGAGTTTAAATATTATCAAATAAGAAAGATACCATTTTTTTTATAGAGAATGTGGTGCATCTGATGAGCACCTGAATCTAGTAGTAACCTTATACCGAAGGTCACGAAATTACTTTTTCAAAGCCGTAAATTTTGATAGTTATCACATTTTATCCCT	Deletion of *STE2* with *TRP1* marker
Ste2KO_Trp5	AAAAAGCTTTCCTACATATTCAAGATTTTTTTCTGTGGGTGGAATACTATTTAAGGAGTGCTATTAGTATCTTATTTGACTTCAAAGCAATACGATACCTTTTCTTTTCACCTGCTCTGGCTATAATTATAATTGGTTACTTAAAAATGCACCGTTAAGAACCATATCCAAGAATCAAAACCGTCGCTTTGAGAGAAATC	
pSTE2_PspOMI_5	TACTGCAGGGGCCCATCCAAT	Cloning of *STE2* promoter with flanking PspOMI and XhoI sites
pSTE2_XhoI_3	TGG TAA TCC TCG AGT TTT GAT TCT TGG ATA TG	
STE2_AarI_5	GCC ATG AAC ACC TGC AAC ACC CTA TGT CTG ATG CGG CTC CTT C	Cloning and mutagenesis of *STE2* ORF with flanking AarI sites
STE2_AarI_3	GTTACAGGCACCTGCAACATCGCTCATAAATTATTATTATC	

pSTE2_AarIfix_3	TTATAATTATAGCCAGAGCACGTG AAAAGAAAAGGTATCGT	Removing AarI site in *STE2* promoter
pSTE2_AarIfix_5	ACGATACCTTTTCTTTTCACGTGCT CTGGCTATAATTATAA	
T1_AarI_3	GTTACA GGC ACC TGC AAC ATC GCT CAT AAA TTA TTA TTA TCT TCA G	Truncation of *STE2* to make T1*
T2_AarI_3	GTT ACA GGC ACC TGC AAC ATC GCT TAC TCA GGA TCA TCG TTG TTG ATA CTA TCA	Truncation of *STE2* to make T2*
STE2_AarI_fuse_3	GTTACAGGCACCTGCAACATCGCT CACCTCCTAAATTATTATTATCTTC	Cloning of *STE2* without a STOP codon
STE2_AarI_fuse358_3	GTTACAGGCACCTGCAACATCGCT CACCTCCATCCGATGTTGTTTCCTT	Cloning of T1 mutant without a STOP codon
STE2_AarI_fuse350_3	GTT ACA GGC ACC TGC AAC ATC GCT CAC CTC CCT CAG GAT CAT CGT TGT T	Cloning of T2 mutant without a STOP codon
SST2_KO_URA_5	GAATTGTTGTCTGTCCTGTTATATT TATACCGGTGAATGAAATCATAAT CCCAGCTCTGTCTTTACTTCTCATC GTCCTTTAACTTTGGAGGTGTTACT GTCGTACGTTCCTTCTAGGTTTTGC ACGCACTATCTGAGGCGTTATAGG TTCAATTTGGTAATTAAAGATAGA GTTGTAAGTTCTAGACACCTGCAA CATG	Deletion of *SST2* with *URA3* marker
SST2_KO_URA_3	AAATGGCTTTATTTAATCATATTAT AGTTGATATAACAAATCACACCTC CGCTTAATTTATACGTAATAGTATT TACAGTCCTTATGGGTATCAAGAC ATATTAGGTTAGGAGAACTAAAGA AAAAAAAAAGGACTGTTTGTGCAA TTGTACCTGAAGATGAGTAAGACT CTCAATGAAATCGATGAATTCGAG CTCGTT	
SST2_BamHI_3	TGG TAA TCG GAT CCT TAG CAC TTT TCT TGG ATT TC	Cloning of *SST2* with flanking PspOMI and BamHI sites
SST2_PspOMI_5	TAC TGC AGG GGC CCT GTC TAC TTC AAA TTC GAA C	
SST2_Q304N_SDM_3	TCA GTA CAG TCC ATT ATC CAA TTC CAT ATA GCC TTT GTT GTA A	Site-directed mutagesis of *SST2*

SST2_Q304N_SDM_5	TTA CAA CAA AGG CTA TAT GGA ATT GGA TAA TGG ACT GTA CTG A	to add Q304N mutation

Supplementary Table 7: Plasmids used in this study

Name	Description
**pRS313**	CEN *HIS3* vector
**pRS315**	CEN *LEU2* vector
**pRS-PSTE2**	CEN *HIS3 STE2* promoter (P_{STE2})
**pRS-STE2**	CEN *HIS3* P_{STE2} *STE2*
**pRS-PADH1**	CEN *HIS3 ADH1* promoter (P_{ADH1})
**pRS-PADH1-STE2**	CEN *HIS3* $PADH1_1$ *STE2*
**pRS-PSTE2-GFP**	CEN *HIS3* P_{STE2} *GFP*
**pRS-PSTE2-CVen**	CEN *HIS3* P_{STE2} C-Venus fragment
**pRS-PSTE2-7KtoR**	CEN *HIS3* P_{STE2} *STE2* 7KtoR variant
**pRS-SST2**	CEN *LEU2* P_{SST2} *SST2*
**pRS-SST2 Q304N**	CEN *LEU2* P_{SST2} $SST2^{Q304N}$
**pRS-PADH1-SST2-NVen**	CEN *LEU2* P_{SST2} *SST2* N-Venus fragment
**pRS-PADH1-SST2 Q304N-NVen**	CEN *LEU2* P_{SST2} $SST2^{Q304N}$ N-Venus fragment

Supplementary Table 8: α-factor pheromones and their peptide sequences.

Species	Peptide sequence
Saccharomyces cerevisiae	WHWLQLKPGQPMY
Naumovozyma castellii	WHWLRLDPGQPLY
Kluyveromyces lactis	WSWITLRPGQPIF
Candida glabrata	WHWVRLRKGQGLF

SUPPLEMENTARY NOTE

Supplementary Note 1: Mathematical model of Ste2 signaling and endocytosis

We investigated the contribution of receptor internalization on mating pathway sensitivity to explain our dose-response data with endocytosis-defective mutants. Ste2 endocytosis involves two distinct processes: constitutive (slow) and ligand-induced (fast). We postulated that the two processes might not contribute equally to sensitivity due to the differences in their rates. However, we faced difficulties in trying to untangle the two phenomena, as the same key amino acid residues operate in both. As such, we resorted to mathematical modeling in order to reveal the contribution of each process.

Our model describes a simplified Ste2 signalling pathway (Supplementary Fig. 3A). In this pathway, a free receptor and ligand associate according to classical single-site kinetics, forming a receptor-ligand complex. Free and bound receptors are internalized at different rates, and the MAP kinase cascade is "black-boxed", such that free and bound receptors signal by directly promoting the expression of GFP through a mating-responsive promoter. GFP is then degraded and diluted by cell division over time. For simplicity and to focus on receptor internalization, we did not include the RGS Sst2. The effects of Sst2 on sensitivity and maximum response were modeled previously [1].

From this model, we obtained the following simplified equation describing the concentration of GFP at steady-state (see derivation below).

$$GFP_{SS} = \tau \frac{k_{Rp}}{k_{Re}} \left(\left(k_{RLs} \frac{k_{Re}}{k_{RLe}} - k_{Rs} \right) \frac{[L]}{[L] + K_D \frac{k_{Re}}{k_{RLe}}} + k_{Rs} \right) \quad (9)$$

As expected, the relationship between GFP and ligand has the form of a Hill equation resulting in a sigmoidal dose-response relationship. As the equation demonstrates, the sensitivity of the response is determined by the product of the dissociation constant K_D and the ratio of the endocytosis rate constants, k_{Re} and k_{RLe}. This ratio implies that the two endocytic rates contribute to sensitivity in an opposing manner; slower basal endocytosis is predicted to improve sensitivity, while slower induced endocytosis has the reverse effect. Conceptually, this is because basal and induced endocytosis have opposite consequences: the former reduces the pool of free receptors while the latter reduces the pool of bound receptors (Supplementary Fig. 3B). This parallels the outcomes of ligand binding and dissociation respectively. As such, the difference between k_{Re} and k_{RLe} modifies the equilibrium constant of the response, thereby controlling sensitivity, much like the difference between k_{on} and k_{off}.

Furthermore, the amount of receptors on the cell surface is given by the ratio k_{Rp} / k_{Re} and is predicted to control the magnitude of the response but not the sensitivity. Although our data confirms the lack of a relationship between receptor overexpression and sensitivity, we observed no effect on magnitude (Figure 4A). This may be because the G protein is limiting in the Ste2 signaling process [2, 3].

We proceeded to perform time simulations in MATLAB (Mathworks) to test whether altering individual endocytic rates could improve mating pathway sensitivity to *Klac* pheromone. Parameter values were obtained from the literature or derived from our data

(Supplementary Table 4). Simulations began 1000 minutes prior to pheromone addition to ensure steady-state concentrations of free receptor and GFP. Following pheromone addition, the concentration of GFP was extracted after 3 hours to construct a dose-response. In this way, we first examine the effects of altering the rates of receptor endocytosis on response strength and sensitivity (Supplementary Fig. 3C). While lowering both rates has the potential to increase response strength, sensitivity moves in the opposite direction. This is reflected when lowering both rates to the same extent as our truncation mutants (Supplementary Fig. 3D). While the lower rate of basal endocytosis improves sensitivity, the lower rate of induced endocytosis has the opposite effect: lowering both leads to greater response strength, but a largely unchanged sensitivity. These simulations help to explain why a global impairment in receptor endocytosis has only marginal effects on sensitivity.

Derivation of steady-state equation

The steady-state equation of GFP concentration was derived from a system of three ordinary differential equations (ODEs) describing changes in the concentration of GFP, free receptor (*R*) and bound receptor (*RL*). The concentration of ligand (*L*) was assumed to be constant throughout (in excess). Furthermore, the model assumes that all chemical species are spatially homogeneous and that changes in concentration are continuous and not significantly affected by discrete molecular events.

$$\frac{d[GFP]}{dt} = k_{RLs}[RL] + k_{Rs}[R] - \frac{[GFP]}{\tau} \tag{1}$$

$$\frac{d[RL]}{dt} = k_{on}[R][L] - k_{off}[RL] - k_{RLe}[RL] \tag{2}$$

$$\frac{d[R]}{dt} = k_{Rp} - k_{on}[R][L] + k_{off}[RL] - k_{Re}[R] \qquad (3)$$

For a more meaningful derivation, we introduce a redundant ODE describing the change in R_T, the total number of surface receptors such that $R_T = R + RL$:

$$\frac{d[R_T]}{dt} = k_{Rp} - k_{Re}[R] - k_{RLe}[RL] \qquad (4)$$

At steady state, the net change in concentration of a species is zero, yielding steady-state concentrations (subscript SS):

$$GFP_{SS} = \tau((k_{RLs} - k_{Rs})RL_{SS} + k_{Rs} \cdot R_{T\,SS}) \qquad (5)$$

$$RL_{SS} = \frac{R_{T.SS}[L]}{[L] + (k_{off} + k_{RLe})/k_{on}} \qquad (6)$$

$$R_{T\,SS} = \frac{k_{Rp}/k_{Re}}{1 + \frac{k_{RLe} - k_{Re}}{k_{Re}} \cdot \frac{[L]}{[L] + K_D + k_{RLe}/k_{on}}} \qquad (7)$$

Substituting the steady-states expressions for free and bound receptors into the equation for GFP_{SS}, we obtain:

$$GFP_{SS} = \tau \frac{k_{Rp}}{k_{Re}} \left(\left(k_{RLs} \frac{k_{Re}}{k_{RLe}} - k_{Rs}\right) \frac{[L]}{[L] + \frac{k_{off} + k_{RLe}}{k_{on}} \frac{k_{Re}}{k_{RLe}}} + k_{Rs}\right) \qquad (8)$$

We can approximate $k_{off} + k_{RLe} \approx k_{off}$, such that the dissociation constant $K_D = k_{off} / k_{on}$ can be used, yielding equation 9. This approximation is reasonable at wild-type values of k_{off} and k_{RLe}, and mutants are expected to have lower values of k_{RLe}.

SUPPLEMENTARY METHODS

Strains and growth conditions

The *S. cerevisiae* strains used in this study were derived from strain W303 and are listed in Supplementary Table 5. Gene deletions were done by standard homologous recombination. For all assays, yeast strains were first transformed with plasmids by the lithium acetate / polyethylene glycol method [10] and grown on selective synthetic defined (SD) plates. Transformed colonies were picked and grown overnight in selective liquid SD medium in a 30°C shaking incubator. Cultures were then diluted to an OD = 0.1 and grown to exponential phase (OD = 0.5 to 0.8) before all treatments. Liquid SD medium was prepared from 6.74 g/L of yeast nitrogen base with ammonium sulfate without amino acids (BioShop), 1 g/L of the appropriate amino acid drop-out mix (BioShop) and 2% v/v glucose. Solid SD medium was prepared by adding 20 g/L of agar to liquid SD.

Plasmid propagation and construction

Plasmid propagation in *Escherichia coli* DH5α was done with Luria Bertani (LB) medium supplemented with 50 μg/mL carbenicillin, and cultures were grown at 37°C. For yeast expression of Ste2 and Sst2, the promoters and ORFs were amplified from yeast genomic DNA (Invitrogen). For Ste2, the promoter and ORF were cloned separately into a variant of the pRS313 vector with designed AarI sites [11]. The promoter was cloned first at the PspOMI and XhoI sites, and its endogenous AarI recognition site was removed by substituting C for G at position -69, generating pRS-PSTE2. The *STE2* ORF was subsequently cloned at the AarI sites, generating pRS-STE2. pRS-PADH1 and pRS-PADH1-STE2 were cloned as described for pRS-PSTE2 and pRS-STE2

respectively, though no AarI recognition site was present in the promoter. The ORF of the endocytosis-defective Ste2 mutant 7KtoR was obtained by gene synthesis (Integrated DNA Technologies) and cloned into pRS-PSTE2 at the AarI sites, generating pRS-PSTE2-7KtoR.

For the expression of Sst2, the *SST2* promoter and ORF were cloned in a single step at the PspOMI and BamHI sites of pRS315, generating pRS-SST2. The mutation Q304N was added with the Quikchange II site-directed mutagenesis kit (Agilent).

For receptor-GFP or fusions, the GFP ORF was first cloned into pRS-pSTE2 at the BamHI site to generate pRS-PSTE2-GFP. This vector was used to accept wild-type or mutant *STE2* ORFs lacking STOP codons at the AarI sites. Similar steps were performed to fuse receptors to C-Venus fragments and Sst2 to N-Venus fragments.

All oligonucleotides and plasmids used in this study are listed in Supplementary Tables 6 and 7 respectively.

Peptide preparations

Peptides were synthesized externally (Biomatik) and dissolved in dimethyl sulfoxide (DMSO) to a stock concentration of 10 mM. For microscopy, peptides were dissolved in water to preserve good optical performance. Peptides are listed in Supplementary Table 8.

Live cell imaging

For shmooing experiments, yeast cells were fixed with 100 µg/mL concavalin A in a 96-well glass bottom Sensoplate (Greiner). The cells were treated with 5 µM of pheromone

and incubated at 30°C for 2 hours. Multiple fields from duplicate samples were imaged using a TCS SP8 confocal microscope (Leica). For visualizing endocytosis, cells expressing GFP-tagged Ste2 receptors were loaded in a CellASIC Onix microfluidic plate (Merck Millipore) and treated with 5 μM of pheromone. A single field from duplicate samples were imaged at intervals up to 45 minutes after treatment.

SUPPLEMENTARY REFERENCES

1. Hao N, Yildirim N, Wang YQ, Elston TC, Dohlman HG. Regulators of G protein signaling and transient activation of signaling - Experimental and computational analysis reveals negative and positive feedback controls on G protein activity. *J Biol Chem* **278**, 46506-46515 (2003).

2. Dosil M, Giot L, Davis C, Konopka JB. Dominant-negative mutations in the G-protein-coupled alpha-factor receptor map to the extracellular ends of the transmembrane segments. *Molecular and cellular biology* **18**, 5981-5991 (1998).

3. Thomson TM*, et al.* Scaffold number in yeast signaling system sets tradeoff between system output and dynamic range. *Proceedings of the National Academy of Sciences of the United States of America* **108**, 20265-20270 (2011).

4. Jenness DD, Spatrick P. Down regulation of the α-factor pheromone receptor in S. cerevisiae. *Cell* **46**, 345-353.

5. Weiner JL, Guttierezsteil C, Blumer KJ. Disruption of Receptor-G Protein Coupling in Yeast Promotes the Function of an Sst2-Dependent Adaptation Pathway. *J Biol Chem* **268**, 8070-8077 (1993).

6. Jorgensen P, Nishikawa JL, Breitkreutz BJ, Tyers M. Systematic identification of pathways that couple cell growth and division in yeast. *Science* **297**, 395-400 (2002).

7. Yi TM, Kitano H, Simon MI. A quantitative characterization of the yeast heterotrimeric G protein cycle. *Proceedings of the National Academy of Sciences of the United States of America* **100**, 10764-10769 (2003).

8. Bashor CJ, Helman NC, Yan SD, Lim WA. Using engineered scaffold interactions to reshape MAP kinase pathway signaling dynamics. *Science* **319**, 1539-1543 (2008).

9. Prilusky J*, et al.* FoldIndex((c)): a simple tool to predict whether a given protein sequence is intrinsically unfolded. *Bioinformatics* **21**, 3435-3438 (2005).

10. Gietz RD, Woods RA. Transformation of yeast by lithium acetate/single-stranded carrier DNA/polyethylene glycol method. *Method Enzymol* **350**, 87-96 (2002).

11. Peisajovich SG, Garbarino JE, Wei P, Lim WA. Rapid Diversification of Cell Signaling Phenotypes by Modular Domain Recombination. *Science* **328**, 368-372 (2010).
